# Large-scale and high-resolution mass spectrometry-based proteomics defines molecular subtypes of nasopharyngeal carcinoma for therapeutic targeting

**DOI:** 10.1038/s41392-026-02742-0

**Published:** 2026-06-23

**Authors:** Yi-Ping Wu, Yao-Hui He, Guo-Sheng Hu, Zhi-Qin Li, Qing-Wen Li, Xiao-Tong Chen, Hui-Ying Ling, Wen Liu, Qin Lin

**Affiliations:** 1 Department of Radiation Oncology, Xiamen Key Laboratory of Radiation Oncology, The First Affiliated Hospital of Xiamen University, School of Medicine, Xiamen University, Xiamen, Fujian China; 2 Xiamen Cancer Center, The First Affiliated Hospital of Xiamen University, Xiamen, Fujian China; 3 State Key Laboratory of Vaccines for Infectious Diseases, Xiang An Biomedicine Laboratory, School of Pharmaceutical Sciences, Xiamen University, Xiang’an South Road, Xiamen, Fujian China; 4 Fujian Provincial Key Laboratory of Innovative Drug Target Research, School of Pharmaceutical Sciences, Xiamen University, Xiang’an South Road, Xiamen, Fujian China; 5 Institute for Future Sciences, University of South China, Changsha, Hunan China; 6 Biomedical Research Center of South China, College of Life Sciences, Fujian Normal University, Fuzhou, China; 7 Shenzhen Research Institute of Xiamen University, Shenzhen, Guangdong China

**Keywords:** Head and neck cancer, Cancer therapy, Tumour biomarkers

## Abstract

Challenges in the precise diagnosis and treatment of nasopharyngeal carcinoma (NPC) remain, mainly due to the absence of a multi-omics-based molecular classification and effective targeted therapies. In this study, we performed proteomic and phosphoproteomic analyses of NPC and non-cancerous nasopharyngeal tissues to identify key dysregulated proteins and phosphorylation networks. Based on these profiles, we classified NPC into two distinct molecular subtypes: S1 and S2, which exhibit significant clinical heterogeneity. Notably, the S2 subtype displayed stronger immune suppressive characteristics. By leveraging proteomic data from both cancerous and non-cancerous tissues, as well as from the two molecular subtypes, we developed robust diagnostic and prognostic models. Through computational drug repurposing and experimental validation, we identified Panobinostat, a pan-histone deacetylase inhibitor, as a potent anti-tumor agent for NPC, demonstrating efficacy in both in vitro and in vivo models. Mechanistically, Panobinostat inhibits MYC expression, thereby suppressing the transcriptional activation of key components in the homologous recombination (HR) DNA repair pathway. This reduction in transcriptional activation impairs HR repair efficiency and leads to the accumulation of DNA double-strand breaks (DSBs). Furthermore, combination therapy with Panobinostat and radiotherapy produced a synergistic effect, significantly enhancing NPC suppression. Additionally, we predicted and validated potential drugs for targeting the S2 subtype of NPC. In conclusion, we identified molecular subtypes of NPC, constructed preliminary diagnostic and prognostic marker panels, and observed the therapeutic potential of Panobinostat, as a monotherapy and in combination with radiotherapy. These findings provide a solid foundation for precision diagnosis, prognostic stratification, and personalized treatment strategies for NPC.

## Introduction

Nasopharyngeal carcinoma (NPC) is a malignant epithelial tumor that arises from the nasopharyngeal mucosal lining and has well-established etiological links to Epstein-Barr virus (EBV) infection.^1,2^ Radiotherapy is the primary treatment for NPC, given its high sensitivity to ionizing radiation. The current paradigm of intensity-modulated radiotherapy (IMRT) combined with platinum-based concurrent chemoradiotherapy has achieved 5-year local control rates exceeding 90% in clinical trials.^3,4^ However, a high proportion of patients present with locally advanced disease at initial diagnosis, leading to approximately 30% experiencing local recurrence and distant metastasis, which ultimately results in treatment failure.^5^

Omics technologies can elucidate molecular mechanisms challenging to address through genomic approaches. Numerous comprehensive genomic profiling studies have identified frequent mutations in several NPC driver genes (e.g., TP53, TRAF3), most of which are located in the NF-κB pathway, underscoring its central role in NPC oncogenesis.^6–10^ These studies also revealed high tumor mutational burden and increased genomic instability in NPC. However, changes in DNA and transcript levels poorly correlate with protein expression.^11^ Post-translational modifications (PTMs) such as phosphorylation generate functionally specific proteoforms, enabling proteomic and phosphoproteomic analyses to provide deeper insights beyond genomics and open new avenues for cancer diagnosis and treatment.^12^ Large-scale proteomic studies have revealed new characteristics and targets in various cancers.^13–19^ Recent small-scale proteomic studies on NPC have provided valuable insights into potential diagnostic biomarkers and therapeutic targets, but their limited sample sizes reduce statistical power for molecular subtyping and may miss important biological features. Specifically, one prior study included only 30 primary NPC samples and 22 control samples, while another enrolled 70 cases but used the 2-D DIGE method, identifying merely 26 differential proteins.^20,21^ Thus, further supplementation and in-depth exploration are still needed in the field of NPC proteomic research.

To address these limitations, we conducted proteomic and phosphoproteomic analyses on 87 NPC samples and 56 non-cancerous samples. This approach enabled us to systematically characterize proteins dysregulation, alterations in phosphorylation sites, and abnormal activation of signaling pathways in NPC. We further classified NPC into two molecular subtypes, subtype 1 and 2, identified distinct protein signatures in both non-cancerous and cancerous tissues, as well as between the two molecular subtypes, and developed diagnostic and prognostic models based on these findings. Through computational drug prediction, we identified several candidate compounds with specific targeting potential against NPC and the S2 subtype, validated their therapeutic efficacy, and explored the molecular mechanisms of the most effective drug and combined treatment strategies.

This study presents, to our knowledge, the largest proteomic and phosphoproteomic dataset for NPC to date. By integrating multi-omics profiling with molecular subtyping, diagnostic model development, and functional drug validation, our work establishes a comprehensive molecular framework that advances the understanding of NPC biology and provides actionable insights for precision diagnosis, prognostic stratification, and personalized treatment strategies.

## Results

### Large-scale proteomic and phosphoproteomic profiling of NPC revealed dysregulated proteins, phosphorylation sites, and signaling pathways in NPC

**Fig. 1:**
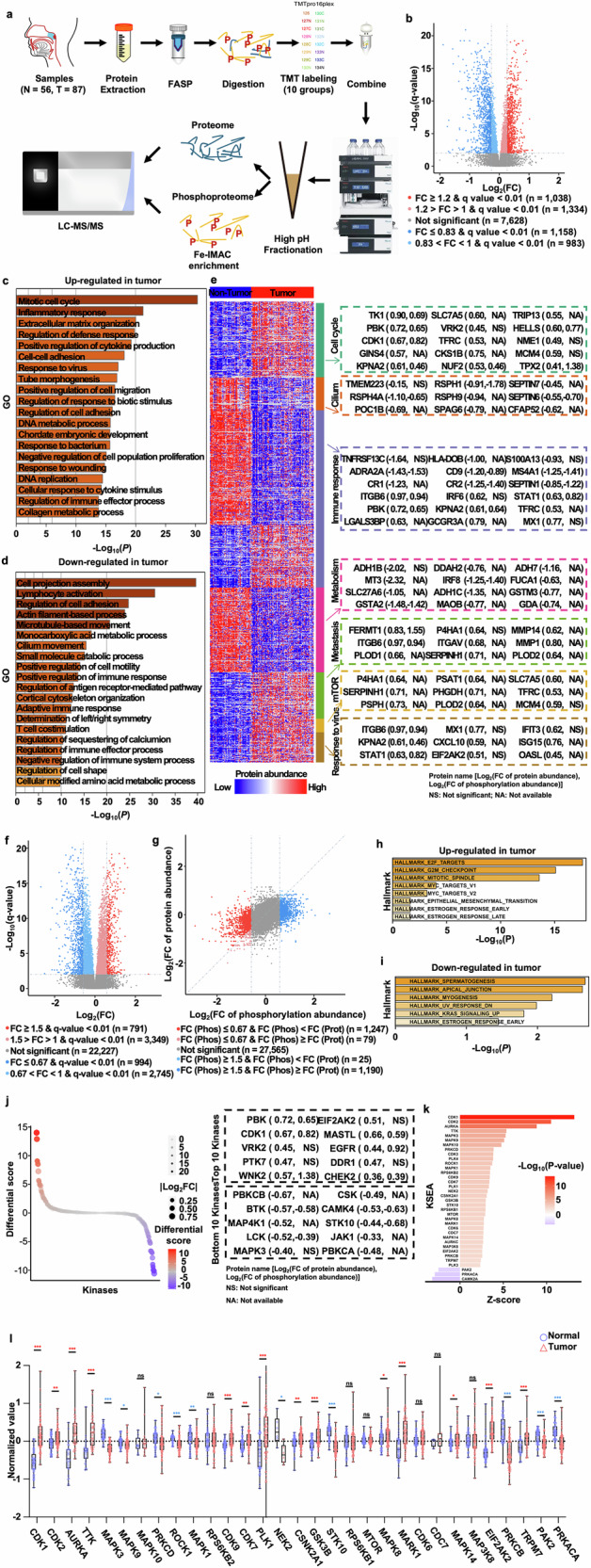
Large-scale proteomic and phosphoproteomic analysis of NPC samples revealed dysregulated proteins, phosphorylation sites, and signaling pathways in NPC. **a** Workflow of NPC (T, *n* = 87) and non-cancerous nasopharyngeal (N, *n* = 56) tissue samples for proteomic and phosphoproteomic analysis. Samples were divided into 10 groups for TMT-based proteomics and phosphoproteomics. **b** Volcano plot showing proteins upregulated or downregulated in tumors. Light red and light blue colors represent proteins with Benjamini-Hochberg (BH) adjusted *P* value < 0.01 and 0.83 < fold change (FC) > 1.2, whereas dark red and dark blue represent proteins with BH adjusted *P* value < 0.01 and FC ≥ 1.2 or FC ≤ 0.83. The remaining proteins are depicted in gray. *P* values were calculated using the two-sided Wilcoxon signed-rank test. Enriched Gene Ontology (GO) Biological Processes for upregulated (**c**) and downregulated (**d**) proteins in tumor samples as shown in (**b**). **e** Heatmap demonstrates the expression levels of differentially expressed proteins (BH adjusted *P* value < 0.01, FC ≥ 1.2 or ≤ 0.83) between tumor and non-tumor samples. The middle panel shows the functions to which the differentially expressed proteins contribute. Scored by protein level and phosphorylation level of the selected proteins, the graph on the right panel shows the proteins that scored high in each functional category between tumor and non-tumor samples. **f** Volcano plot showing phosphosites upregulated or downregulated in tumors. Light red and light blue colors represent proteins with BH adjusted *P* value < 0.01, whereas dark red and dark blue represent proteins with BH adjusted *P* value < 0.01 and fold change (FC) ≥ 1.5 or fold change (FC) ≤ 0.67. The remaining proteins are depicted in gray. *P* values were calculated using the two-sided Wilcoxon signed-rank test. (**g**) Comparison of the alterations of phosphosite abundance (FC.Phos) with those of the corresponding protein abundance (FC.Prot). Light red colors indicate a significant downregulation of phosphosites (BH adjusted *P* value < 0.01 and FC.Phos ≤ 0.67), whereas red further requires FC.Phos < FC.Prot value. Light blue colors indicate significantly upregulated phosphosites (BH adjusted *P* value < 0.01 and FC.Phos ≥1.5), whereas blue further requires FC.Phos > FC.Prot. The remaining phosphosites are displayed in gray. *P* values were calculated using the two-sided Wilcoxon signed-rank test. Enriched Hallmark get sets for upregulated (**h**) and downregulated (**i**) phosphosites in tumor samples as shown in (**f**). **j** The kinases were scored based on the kinmap database, and the table on the right shows the top 10 and bottom 10 scoring kinases. *P* values (permutation test) are represented by the size of each circle. The color gradient indicates the level of score. **k** Enriched kinases determined by KSEA analysis. Red and purple bars represent kinases with increased and decreased activity in tumor, respectively. *P* values (permutation test) are represented by the color gradient. **l** The expression levels of the kinases as shown in (**k**) between NPC tumor and non-tumor tissues (Tumor, *n* = 79; Non-tumor, *n* = 52) by proteomic data. *P* values were calculated using the two-sided Wilcoxon signed-rank test. (Red*: up-regulated in NPC tumor, blue*: down-regulated in NPC tumor; *P < 0.05, **P < 0.01, ***P < 0.001)

We performed isobaric tandem mass tag (TMT)-based proteomic analysis on 87 NPC tissue samples and 56 non-cancerous nasopharyngeal tissue samples (Fig. 1a, Fig. S1a, and Data S1). A total of 12,141 distinct proteins were identified, with an average of 9201 proteins quantified per group (Fig. S1b and S1c, and Data S2). Of these, 9016 proteins were quantifiable in at least half of the samples, and 6,951 proteins were detected across all samples (Fig. S1b). In addition to proteome analysis, we conducted TMT-based phosphoproteomic profiling on the same samples to capture phosphorylation events (Fig. 1a).^22^ This analysis identified 6066 phosphoproteins with 30,106 phosphosites, averaging 16,256 phosphosites per group (Fig. S1d and S1e, and Data S3). Of these phosphosites, 15,028 phosphosites (49.9%) were quantifiable in at least half of the samples, and 7881 (26.2%) were detected across all samples (Fig. S1d). Integrative analysis revealed 5409 phosphoproteins (89.2% of the total phosphoproteome) with matched proteomic quantification (Fig. S1f). Twelve samples, 8 NPC tissue and 4 non-cancerous nasopharyngeal tissue samples, were excluded due to issues identified in the subsequent clustering analysis, and downstream analyses were conducted on the rest 79 NPC and 52 non-cancerous nasopharyngeal tissue samples. Principal component analysis (PCA) and unsupervised hierarchical clustering clearly distinguished tumor and non-tumor proteomic profiles, with no batch effects observed between the groups (Fig. S1g-S1j).

To delineate the NPC-specific proteomic landscape, we performed comparative analysis between tumor and non-tumor samples. Of the 12,141 proteins quantifiable in at least one of the 10 groups (Fig. S1b), 1038 proteins were up-regulated, and 1158 proteins were down-regulated in tumor samples compared to non-tumor samples (Fig. 1b and Data S4). Gene Ontology (GO) enrichment analysis revealed that up-regulated proteins in tumor samples were significantly enriched in cell cycle regulation, extracellular matrix remodeling processes, and immune responses, among others (Fig. 1c and Data S4). Conversely, down-regulated proteins in tumor samples were primarily associated with lymphocyte activation, adaptive immune functions, metabolic pathways, and cilium movement, among others (Fig. 1d and Data S4). Integrated analysis further identified differentially expressed genes enriched in seven functional clusters: cell cycle, cilium function, immune responses, metabolism, metastasis, mTOR signaling, and responses to virus (Fig. 1e and Data S5). Notably, multiple interferon-stimulated genes (ISGs), including STAT1,^23^ MX1,^24^ CXCL10,^25^ IFIT3,^26^ ISG15,^27^ and OASL,^28^ were significantly up-regulated in tumor versus non-tumor samples. These genes were functionally enriched in immune responses and anti-viral pathways.^29^ This observation aligns with prior reports of elevated ISG expression in NPC epithelial cells,^30^ a phenomenon potentially linked to the prevalence of non-keratinizing EBV-associated subtypes in Chinese populations.^1,31^ EBV-driven chronic inflammation and viral persistence are well-established contributors to NPC pathogenesis.^32^ Interestingly, specific ISGs (e.g., STAT1, MX1, IFIT3) exhibit dual functions: while mediating antiviral responses, they may also promote DNA damage resistance.^33–35^ Given the central role of radiotherapy in NPC management,^3^ these ISGs may contribute to therapeutic resistance and negatively affect patient prognosis.

Following the characterization of dysregulated proteins, we assessed the differences in phosphosites abundance between tumor and non-tumor samples. Of the 30,106 phosphosites quantifiable in at least one of the 10 groups, 1,785 phosphosites were significantly dysregulated in tumors compared to non-tumor samples, with 791 up-regulated and 994 down-regulated sites in tumors (Fig. 1f and Data S4). Notably, the fold-change (FC) in phosphorylation abundance for most phosphosites was greater than that of the corresponding protein abundance in NPC (Fig. 1g). Hallmark enrichment analysis of differentially phosphosites revealed tumor-specific activation of cell cycle and epithelial-to-mesenchymal transition (EMT) pathways, contrasted by elevated UV responses and KRAS signaling in non-tumor samples (Fig. 1h, i, and Data S4). Systematic kinase profiling assessed protein abundance across samples and identifying PBK, CDK1, VRK2, PTK2, WNK2, EIF2AK2, MASTL, EGFR, DDR1, and CHEK2 as the top 10 highly expressed kinases in tumors (Fig. 1j). Notably, most of these kinases also displayed enhanced phosphorylation patterns, with CDK1, WNK2, and EGFR showing more pronounced phosphorylation changes relative to protein abundance, suggesting concurrent overexpression and activation. Similarly, the bottom 10 expressed kinases, including PBKCB, BTK, MAP4K1, LCK, MAPK3, CSK, CAMK4, STK10, JAK1, and PBKCA, exhibited reduced phosphorylation (Fig. 1j). We further assessed kinase activity using Kinase Substrate Enrichment Analysis (KSEA),^36^ with CDK1, CDK2, and AURKA showing the highest scores (Fig. 1k). CDK1 and CDK2 are classic regulators of the cell cycle, and their dysregulation is associated with chromosomal instability and disruptions in the S phase and G2/M transition—key events in tumorigenesis.^37^ These findings mechanistically explain the observation that cell cycle genes are enriched in dysregulated proteins in tumor samples. Conversely, PAK2, PRKACA, and CAMK2A exhibited negative scores, suggesting their potential roles in anti-cancer processes in NPC. The expression of these kinases was also examined based on proteomic analysis of tumor and non-tumor tissues, revealing that the majority of active kinases were significantly up-regulated, while kinases with decreased activity were down-regulated (Fig. 1l).

Given the close association between EBV infection and the development of NPC, particularly WHO type III NPC, we analyzed differentially expressed proteins between EBV-positive and EBV-negative NPC patients. A total of 90 molecules were upregulated in the EBV-positive group, primarily enriched in B-cell receptor-related pathways and the mTOR signaling pathway (Figs. S1k and S1l). This aligns with the well-established fact that EBV infection is closely linked to B cells.^38^ Additionally, EBV infection promoting mTOR signaling activation has been previously reported in Burkitt lymphoma.^39^ In contrast, 65 proteins were downregulated in the EBV-positive group, mainly involved in functions such as ECM receptor interaction (Figs. S1k and S1m). However, it should be noted that among our NPC patients, only 12 were confirmed as EBV-negative, while 52 were EBV-positive. Due to the relatively small sample size of the EBV-negative group, the significance of differential genes between EBV-positive and EBV-negative NPC was determined solely based on *p* values. Therefore, these findings require further analysis and validation in future studies.

### Diagnostic signatures were identified in NPC

**Fig. 2:**
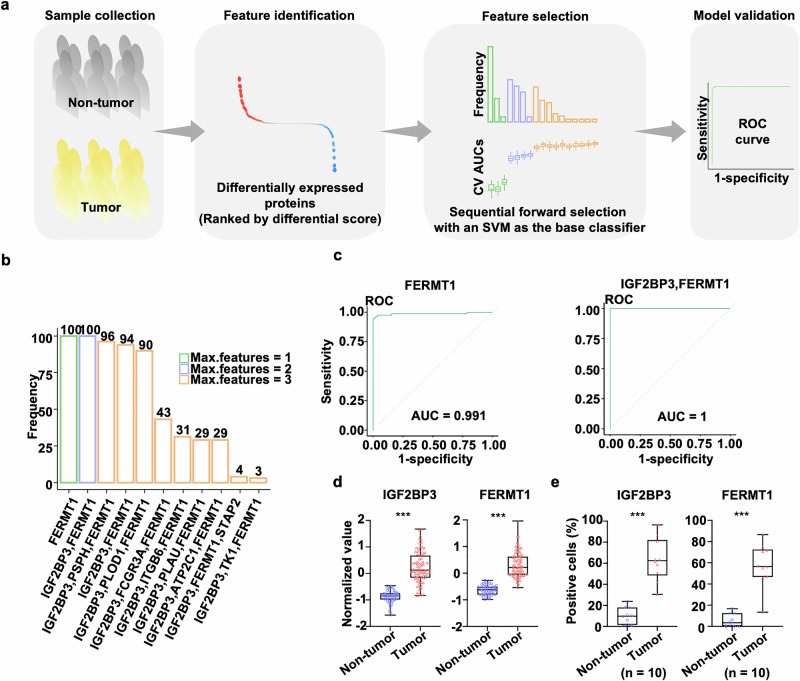
Diagnostic signatures were identified in NPC. **a** Workflow of identifying diagnostic signatures based on differentially expressed proteins between NPC tumor and non-tumor samples. **b** Bar plots of the frequency of the signatures in 100 times feature selections. Green, purple, and orange indicate that the maximum number of features is 1, 2 and 3, respectively. **c** ROC curves of the Support Vector Machine (SVM) model with signature “FERMT1” (left panel) and “IGF2BP3, FERMT1” (right panel). **d** Box plots of the protein expression of IGF2BP3 and FERMT1 (Tumor, *n* = 79; Non-tumor, *n* = 52) by proteomic data. *P* values are calculated by two-sided Wilcoxon rank-sum test. **e** Ten cases of NPC tissues and non-cancerous nasopharyngeal tissues were subjected to immunostaining analysis using anti-IGF2BP3 and anti-FERMT1 antibody. The number of positive cells was quantitatively analyzed by Qupath. (mean ± SEM; *P < 0.05, **P < 0.01, ***P < 0.001)

The current histopathological diagnosis of NPC, which primarily relies on hematoxylin-eosin staining and immunohistochemical markers (e.g., CK, EBER, and Ki-67), carries a risk of false-negative results that affects a subset of patients, highlighting an urgent need for novel, highly specific diagnostic markers to improve diagnostic accuracy for this cohort. To address the persistent risks of diagnostic inaccuracies, we employed machine learning-based feature selection for protein biomarker discovery (Fig. 2a). Initially, we specified the maximum number of features as 1, 2, or 3 for the feature selection process (Fig. 2b). Using the maximum area under the ROC curve (AUC) as the criterion, 11 unique signatures were identified, among which the signatures “FERMT1” and “IGF2BP3, FERMT1” had the highest frequencies (Fig. 2b). Evaluated via cross-validation on the basis of the internally constructed proteomic training set, the “IGF2BP3, FERMT1” signature demonstrated optimal predictive efficacy, with a mean AUC of 1 (Fig. 2c). Based on the proteomic data from this study, the expression of IGF2BP3 and FERMT1 was significantly higher in tumor samples compared to non-tumor samples (Fig. 2d). IGF2BP3 is an RNA-binding protein that plays a critical role in post-transcriptional regulation, particularly affecting the stability and localization of specific mRNAs and influencing their translation. It is closely linked to processes such as cell growth and proliferation.^40^ FERMT1, also known as Kindlin-1, belongs to the Kindlin family, which is essential for regulating cell adhesion and integrin activation. It modulates integrin activity by interacting with integrin-associated proteins and is crucial for cell attachment to the extracellular matrix and cell motility.^41^ We further validated the elevated expression of IGF2BP3 and FERMT1 in NPC tissues compared to non-cancerous nasopharyngeal tissues using immunofluorescence staining on clinical tissue sections (Fig. 2e).

### Proteomic analysis classifies patients with NPC into two distinct subtypes: S1 and S2

**Fig. 3:**
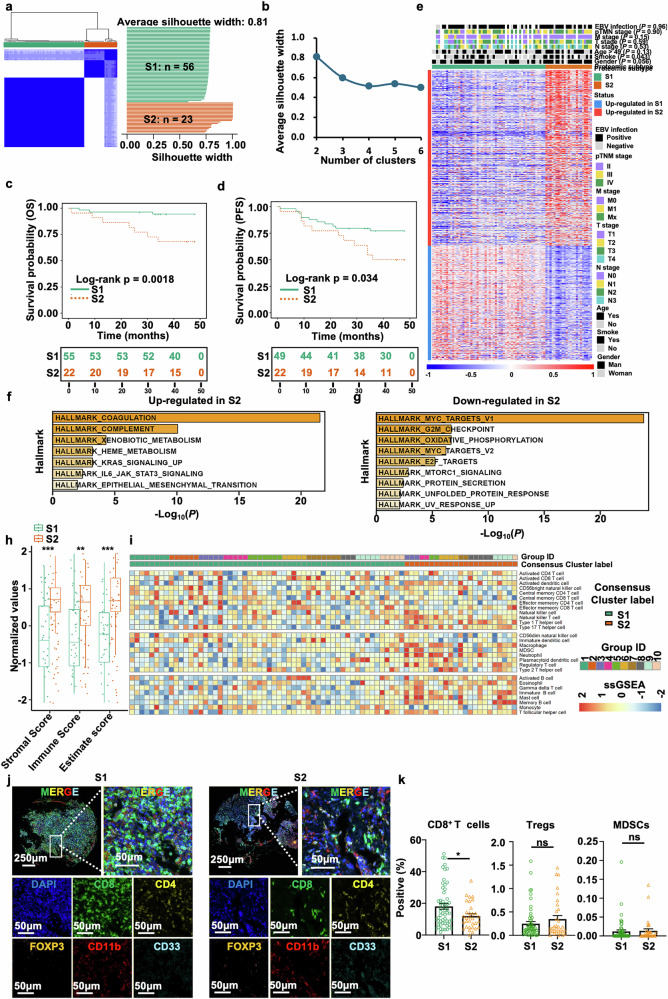
Molecular subtypes of NPC were defined by proteomic analysis. **a** Consensus clustering of NPC tumor samples. The left panel shows consensus matrices of the NPC samples (*n* = 79) with two clusters. Consensus clustering was performed on the top 5000 most-variant proteins in Prot1. The right panel shows the silhouette-width plot. **b** Average silhouette-width plot. The average silhouette width takes the maximum value when number of clusters was 2. **c** Kaplan-Meier curves of overall survival (OS) for subtype S1 and S2. *P* values were calculated by two-sided log-rank test. **d** Kaplan-Meier curves of progression-free survival (PFS) for subtype S1 and S2. *P* values were calculated by two-sided log-rank test. **e** Heatmap representation of the relative protein abundance of differentially expressed proteins between S1 and S2 (BH adjusted *P* < 0.01, FC ≥ 1.2 or ≤0.83). The upper panel shows the association between molecular subtypes and clinicopathologic characteristics. Enriched Hallmark get sets for upregulated (**f**) and downregulated (**g**) proteins in S2 subtype as shown in Fig. S2c. **h** Estimate score and abundance of stromal and immune cells in S1 and S2 subtypes were analyzed by ESTIMATE. *P* values were calculated by two-sided wilcoxon rank-sum test. **i** Single-sample Gene Set Enrichment Analysis (ssGSEA) followed by Pearson’s correlation analysis between immune cell enrichment scores (derived from GSVA) and subtype classifications. Red nodes indicate positive correlation, while blue nodes indicate negative correlation. **j** Multicolor immunofluorescence staining illustrates the immune cells including CD8⁺ T cells, Tregs, and MDSCs in S1 and S2 subtype tissue specimens. Nuclei were visualized with DAPI, while immune subsets were distinguished using CD8⁺ (CD8⁺ T cells), CD4⁺/Foxp3⁺ (Tregs), and CD11b⁺/CD33⁺ (MDSCs). White dashed boxes outline regions shown at higher magnification. Scale bars: 250 μm (overview), 50 μm (magnified views and single-channel panels). **k** Bar graphs quantify the proportions of CD8^+^ T cells, Tregs, and MDSCs in S1 vs. S2 subtypes. Statistical analysis was conducted using the square-root transformed *t* test. (mean ± SEM; *P < 0.05; ns: non-significant)

Current clinical stratification of NPC remains categorized into three major subtypes based on histopathological criteria: keratinizing squamous, non-keratinizing, and basaloid squamous subtypes, among which the non-keratinizing subtype is predominant in the Chinese population. However, this histopathology-based classification system lacks clear prognostic value and definitive therapeutic implications. To address this critical limitation, molecular subtyping of NPC is therefore essential for gaining deeper insights into its biological heterogeneity and clinical relevance. Herein, we aimed to stratify NPC tumor samples using proteomic profiling, with the goal of identifying molecular subtypes characterized by distinct expression signatures. Furthermore, we sought to determine whether these subtypes correlate with prognosis, helping to identify individuals with adverse outcomes in NPC. We assessed the average silhouette width for clustering across 2–6 groups and found that it was maximized with two clusters. We designated these subtypes as S1 and S2 (Fig. 3a, b, and Data S6), which could be clearly differentiated through principal component analysis (Fig. S2a). The S1 and S2 subtypes comprised 56 and 23 tumor samples, respectively (Fig. S2b and Data S6). Patients in the S2 subtype demonstrated poorer overall survival (OS) and progression-free survival (PFS) compared to those in the S1 subtype (Fig. 3c, d). In addition, we performed COX regression analysis to determine whether there were risk factors associated with the subtypes. Apart from smoking history, patients with S1 and S2 subtypes showed no significant differences in other clinical characteristics. (Fig. 3e, Fig. S2b, and Data S6).

Further analysis revealed 1,962 differentially expressed proteins between the S1 and S2 subtypes, with 1187 up-regulated and 774 down-regulated in S2 compared to S1 (Fig. S2c). In-depth analysis showed that proteins up-regulated in the S2 subtype were primarily involved in complement and coagulation cascades, while those down-regulated were clustered in MYC-targets, G2/M checkpoint, and E2F targets (Fig. 3g, h, and Data S6).

We observed that while immune response pathways are broadly enriched in NPC tumor tissues compared to non-tumor tissues, distinct differences in immune pathway activation exist between the two molecular subtypes. Notably, the S2 subtype demonstrated a more pronounced enrichment of immune-related pathways than the S1 subtype. To systematically evaluate immune and stromal cellular infiltration, we applied the ESTIMATE (Estimation of Stromal and Immune cells in Malignant Tumor tissues using Expression data)^42^ algorithm to calculate stromal and immune scores. This analysis revealed higher levels of immune infiltration in the S2 subtype compared to S1 (Fig. 3h). The stromal infiltration scores followed a similar pattern, with S2 exhibiting higher stromal content (Fig. 3h). Consequently, the ESTIMATE score was significantly higher in the S2 subtype, suggesting lower tumor purity compared to the S1 subtype (Fig. 3h). Further characterization of immune cell composition uncovered subtype-specific patterns. While the S2 subtype exhibited robust immune cell infiltration, its microenvironment was dominated by immunosuppressive populations, including myeloid-derived suppressor cells (MDSCs),^43^ regulatory T cells (Tregs), and Th2 cells,^44^ with a notably reduced abundance of activated CD8^+^ T cells. In contrast, the S1 subtype showed a CD8^+^ T cell-enriched immune landscape (Fig. 3i and Fig. S2d). These findings suggest divergent immune evasion mechanisms between the subtypes. The immunosuppressive phenotype of the S2 subtype appeared mechanistically linked to the activation of complement and coagulation cascades. Activation of complement components C3/C5 through their receptors C3AR/C5AR1 promoted recruitment of MDSCs, tumor-associated macrophages (TAMs), and Tregs, thereby suppressing antitumor T cell responses. Complement-mediated induction of matrix metalloproteinases (MMPs) further facilitated tumor progression by enhancing angiogenesis and extracellular matrix remodeling. Supporting this mechanism, we observed elevated expression of C3, C5, C5AR1, and multiple MMPs in the S2 subtype based on our proteomic data (Fig. S2e), indicating that coordinated activation of complement-coagulation pathways may drive both immune suppression and metastatic dissemination in this aggressive subtype. In contrast, the S1 subtype demonstrated prominent enrichment of proliferative signaling pathways. Mechanistically, EIF3J and EIF4F – key eukaryotic translation initiation factors–were significantly enriched, where they regulate ribosome biogenesis and protein synthesis to promote tumor cell proliferation (Fig. S2f). Furthermore, coordinated enrichment of MCM family members in MYC pathways linked DNA replication initiation and elongation processes to aberrant proliferative activity (Fig. S2f). The significantly elevated tumor purity in the S1 subtype may be closely associated with the hyperactivation of autonomous proliferation pathways in tumor cells. To validate the immunosuppressive infiltrative microenvironment of the S2 subtype, we performed multiplex immunofluorescence assays on a tissue microarray— targeting CD8⁺ T cells, Tregs, and MDSCs (Data S6). The results revealed a significant decrease in CD8⁺ T cell infiltration in the S2 subtype, while Tregs and MDSCs showed an increasing trend (Fig. 3j, k).

These findings suggest distinct immune evasion mechanisms across the two subtypes. The immunosuppressive phenotype of the S2 subtype appears mechanistically related to activation of the complement and coagulation cascades. Previous reports indicate that complement components C3 and C5, through receptors C3AR and C5AR1, promote recruitment of MDSCs, tumor-associated macrophages (TAMs), and Tregs,^45,46^ thereby suppressing antitumor T cell responses. Complement-mediated induction of matrix metalloproteinases (MMPs) further enhances tumor progression by promoting angiogenesis and extracellular matrix remodeling.^45^ In support of this proposed mechanism, our proteomic analysis revealed elevated expression of C3, C5, C5AR1, and several MMPs in the S2 subtype (Fig. S2e), indicating that coordinated activation of complement and coagulation pathways may contribute to both immune suppression and metastatic dissemination in this aggressive subtype. In contrast, the S1 subtype demonstrated prominent enrichment of proliferative signaling pathways. We observed significant enrichment of key eukaryotic translation initiation factors EIF3J and EIF4F (Fig. S2f), which are involved in ribosome biogenesis and protein synthesis, potentially promoting tumor cell proliferation.^47,48^ Furthermore, coordinated enrichment of MCM family members^49^ within MYC pathways suggests a link to DNA replication initiation and elongation processes, which may collectively drive aberrant proliferative activity. The significantly elevated tumor purity observed in the S1 subtype may be closely associated with the hyperactivation of such autonomous proliferation pathways within tumor cells.

### Diagnostic signatures were identified for S1 and S2 subtypes in NPC

**Fig. 4:**
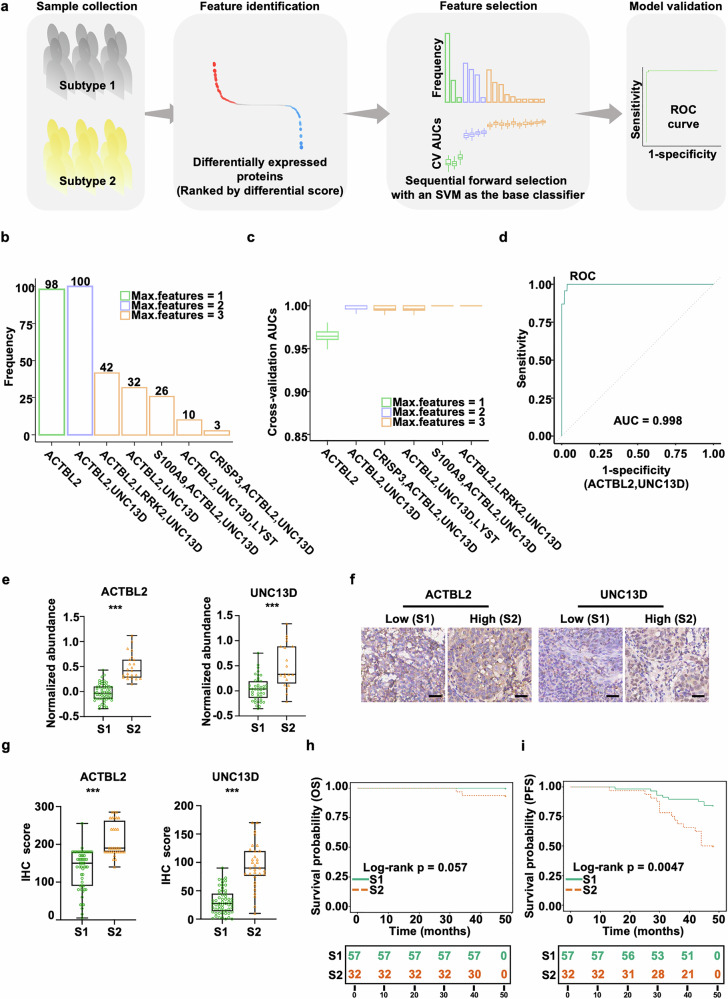
Diagnostic signatures of subtypes were defined by proteomic analysis. **a** Workflow of identifying diagnostic signatures of subtypes in NPC based on differentially expressed proteins. **b** Bar plots of the frequency of the signatures in 100 times feature selections. Green, purple, and orange indicate that the maximum number of features is 1, 2, and 3, respectively. **c** Bar plots of the cross-validation of the signatures in 100 times feature selections. **d** ROC curve of the SVM model with signature 2 by proteomic data. **e** Box plots of the expression of ACTBL2 and UNC13D in S1 and S2 subtypes by proteomic data. *P* values are calculated by two-sided wilcoxon rank-sum test. (***P < 0.001). **f** The expression of ACTBL2 and UNC13D in a NPC tissue microarray cohort (*n* = 89) was examined by immunohistochemistry (IHC) analysis. Representative images are shown. Scale bar, 50 μm. **g** IHC scores for ACTBL2 (left) and UNC13D (right) in patients predicted as S1 (*n* = 57) or S2 (*n* = 32) subtypes in the NPC cohort as described in (**f**). *P* values were calculated using the two-sided Wilcoxon rank-sum test. Kaplan-Meier curves for OS (**h**) and PFS (**i**) of each predicted subtype in the NPC cohort as described in (**a**). *P* values were determined using the two-sided log-rank test. (***P < 0.001)

We next aimed to identify subtype-specific diagnostic signatures with potential clinical applicability. To this end, we performed feature selection with the maximum number of features set to 1, 2 or 3 (Fig. 4a). Using AUC as the criterion, six unique signatures were identified, among which the signature “ACTBL2, UNC13D” appeared most frequently (Fig. 4b). Cross-validation experiments demonstrated that signature “ACTBL2, UNC13D” achieved excellent predictive performance (mean AUC = 0.998) (Fig. 4c, d). The expression of ACTBL2 and UNC13D was higher in the S2 subtype compared to the S1 subtype (Fig. 4e). ACTBL2, a β-actin homolog, promotes tumor cell migration and invasion by regulating cytoskeletal dynamics and EMT.^50^ Its overexpression is linked to metastasis and poor prognosis in cancers such as breast^51^ and ovarian cancer.^50^ UNC13D, involved in vesicle secretion and cytotoxic granule release in lymphocytes, has been shown to promote tumor progression in solid tumors like pancreatic cancer.^52^

To further validate the subtype diagnostic model, we performed quantitative immunohistochemistry (IHC) analysis of ACTBL2 and UNC13D expression in 89 external NPC patient samples (Data S6). Using this model, we predicted 57 patients as the S1 subtype and 32 patients as the S2 subtype (Data S6). IHC results showed that in the S2 subtype, the staining intensity of ACTBL2 and UNC13D was significantly higher than in the S1 subtype (Fig. 4f, g). The subcellular localization of both proteins remained consistent between the two subtypes, with ACTBL2 predominantly localized in the cytoplasm and UNC13D located in both the cytoplasm and nucleus (Fig. 4f). Additionally, Kaplan-Meier curves showed that the S2 subtype had significantly poorer PFS outcomes compared to the S1 subtype, and the S2 subtype also displayed a trend toward worse overall survival (OS), indicating that the subtype diagnostic model we developed can accurately predict NPC subtypes (Fig. 4h, i).

### Drug prediction and validation showed that Panobinostat is potent in suppressing NPC

**Fig. 5:**
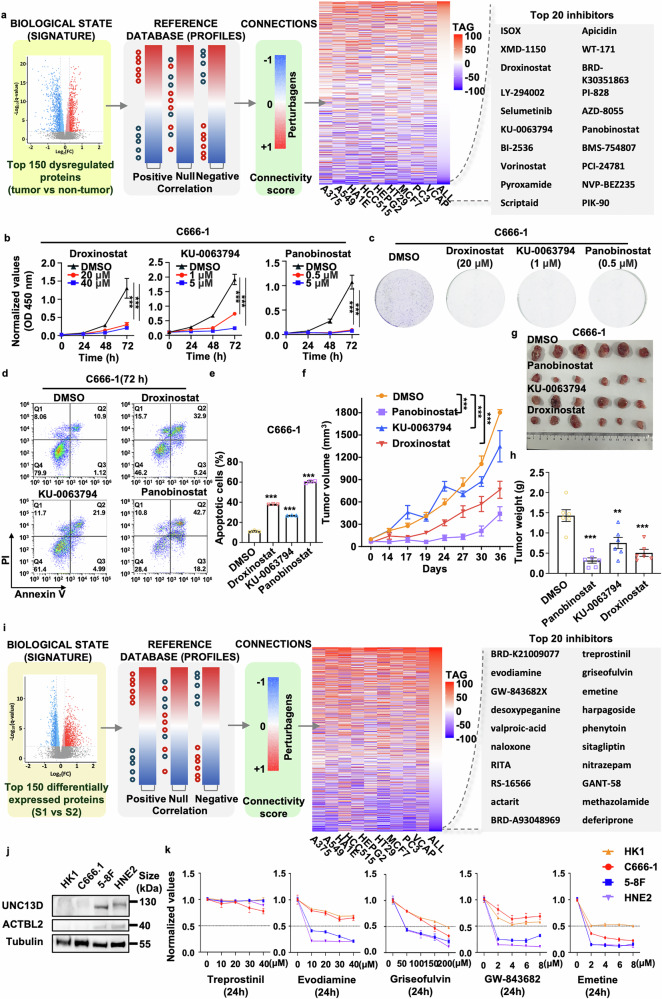
Drug prediction and validation for NPC and S2 subtype. **a** Workflow of drug prediction. The top 150 upregulated and 150 downregulated proteins in tumor tissues were used as the query signature to match the reference profiles of perturbagens in CMAP to calculate connectivity scores. Perturbagens are sorted by connectivity score in increasing order, and the top 20 perturbagens are displayed in the right table. **b** The viability of C666-1 cells was assessed by CCK-8 assay after treatment with three drugs at concentrations as indicated for 0, 24, 48, and 72 h (*n* = 3; mean ± SEM; ***P < 0.001). **c** C666-1 cells treated with DMSO or three drugs (Droxinostat, 20 μM; KU-0063794, 1 μM; Panobinostat, 0.5 μM) were subjected to colony formation assay for 2 weeks. **d** C666-1 cells were treated with DMSO or three drugs (Droxinostat, 20 μM; KU-0063794, 1 μM; Panobinostat, 0.5 μM) for 72 h, followed by staining with Annexin V-FITC and PI. Apoptotic cells were detected by flow cytometry analysis. **e** Quantification of the apoptotic cells in (**d**) is shown. (*n* = 3; mean ± SEM; ***P < 0.001). **f** Female BALB/c nude mice (4 weeks-old) were subcutaneously inoculated with C666-1 cells (5 × 10⁶ cells/mouse) in the right flank. When the tumor volume reached 50 mm³, tumor-bearing mice were randomized into control and experimental groups (*n* = 6 per group). Treatment regimens included intraperitoneal administration of either vehicle control (DMSO) or therapeutic agents (Panobinostat, 10 mg/kg; KU-0063794, 3 mg/kg; Droxinostat, 15 mg/kg) three times per week for four consecutive weeks, continuing until the predefined experimental endpoint. The growth curves of tumors are shown (mean ± SEM; ***P < 0.001). *P* values were calculated by two-way ANOVA. **g** Mice were sacrificed, and tumors were collected and imaged. **h** The weight of tumors as shown in (**g**) is shown (mean ± SEM; **P < 0.01, ***P < 0.001). *P* values were calculated by unparied *t* test. **i** Workflow of drug prediction for Subtype 2. The top 150 differentially expressed proteins between S1 and S2 subtypes were used as the query signature to match the reference profiles of perturbagens in CMAP to calculate connectivity scores. Perturbagens were sorted by connectivity score in increasing order, and the top 20 perturbagens are displayed in the right table. **j** HK1, C666-1, 5–8 F, and HNE2 cells were subjected to IB analysis with antibodies as indicated. **k** The viability of HK1, C666-1, 5–8 F, and HNE2 cells was assessed by CCK-8 assay after treatment with compounds at concentrations as indicated for 24 h (*n* = 3; mean ± SEM)

To identify novel therapeutic candidates for NPC, we analyzed the top 150 differentially expressed proteins between tumor and non-tumor tissues (Fig. 1b and Data S7). We mapped this protein signature to the Connectivity Map (CMAP)^53^ database and calculated connectivity scores to identify compounds capable of reversing the tumor-associated expression profile. Compounds with high negative connectivity scores were prioritized, resulting in 20 candidate drugs (Fig. 5a, Fig. S3a, and Data S7). These drugs represented diverse mechanistic classes, including histone deacetylase (HDAC) inhibitors, leucine-rich repeat kinase (LRRK) inhibitors, APEX nuclease inhibitors, and targeted agents against mTOR, PI3K/AKT, MEK/MAPK, PLK1, and IGF1R signaling axes (Fig. S3a). These drugs target critical oncogenic pathways, engaging epigenetic regulators (HDAC isoforms), DNA damage response mediators (LRRK2, APEX1), metabolic/proliferative signaling hubs (mTOR, PI3K, MAPK), mitotic controllers (PLK1), and growth factor receptors (IGF1R). To validate the efficacy of these compounds in NPC, we excluded drugs previously studied in NPC, those with poor clinical outcomes, and those of unknown identity, leaving three candidates: Droxinostat, KU-0063794, and Panobinostat. Panobinostat and Droxinostat are broad-spectrum HDAC inhibitors with distinct therapeutic applications. Panobinostat exerts antitumor effects through transcriptional modulation and apoptosis induction and has been FDA-approved for the treatment of relapsed or refractory multiple myeloma. In contrast, Droxinostat has shown potential therapeutic efficacy against hematologic malignancies, such as T-cell lymphoma, likely via epigenetic regulation. KU-0063794 is a small-molecule inhibitor targeting the mTOR pathway, thereby suppression cell proliferation and metabolic activity in cancer cells. Preclinical studies have confirmed its antitumor activity in multiple solid tumor models, though it has not yet entered clinical development.

We first evaluated the efficacy of these drugs in two NPC cell lines, C666-1 and HK1, using cell viability assays. All three compounds exhibited significant inhibitory effects on cell proliferation in both cell lines (Fig. 5b and Figs. S3b and S3c). These findings were further supported by colony formation assays (Fig. 5c and Fig. S3d). All compounds significantly induced apoptosis, with Panobinostat showing the most pronounced pro-apoptotic effects in both C666-1 and HK1 cell lines (Fig. 5d, e, and Fig. S3e and S3f). To validate the efficacy of these drugs in vivo, female BALB/c nude mice were subcutaneously inoculated with C666-1 cells, followed by drug administration. The results showed that all three drugs effectively suppressed tumor growth, with Panobinostat showing the most significant effects (Fig. 5f, g). The inhibitory effects on cell proliferation of Panobinostat was confirmed in another two NPC cell lines, 5–8 F and HNE2 (Fig. S3g and S3h). Furthermore, Panobinostat significantly inhibited the viability of NPC organoids (Figs. S3i and S3j).

We further searched for therapeutic targets specific to the S2 subtype. Building on the differential gene expression profiles between the S1 and S2 subtypes, we conducted targeted drug prediction for the S2 subtype (Fig. 5k). The top 20 candidate therapeutic agents identified spanned diverse mechanistic classes, including dual-specificity protein phosphatase inhibitors, prostacyclin analogs, tubulin-targeting agents, and HDAC inhibitors. These compounds target critical molecular targets such as the prostacyclin receptor (PTGIR), transient receptor potential vanilloid 1 (TRPV1), polo-like kinase 1 (PLK1), HDAC1, and murine double minute 2 homolog (MDM2) (Fig. S3k). As for the S1 subtype, the identified top 20 candidate therapeutic drugs include CDK inhibitors, JNK inhibitors, MAPK inhibitors, AKT inhibitors, histone deacetylase (HDAC) inhibitors, etc (Fig. S3i).

Given the poorer prognosis of the S2 subtype, we further investigated the inhibitory effects of the predicted S2 subtype inhibitors on different NPC subtypes. After excluding compounds with unclear information, we selected five candidates—Treprostinil, Evodiamine, Griseofulvin, GW-843682, and Emetine—for subsequent validation. We first examined the protein expression levels of ACTBL2 and UNC13D in four NPC cell lines: HK1, C666-1, 5–8 F, and HNE2. ACTBL2 and UNC13D were found to be highly expressed in 5–8 F and HNE2 cells, while expression was nearly undetectable in HK1 and C666-1 cells (Fig. 5k). Then, we investigated the effects of the five compounds on cell viability of these cell lines and found that, except for Treprostinil, the other four compounds exhibited higher cytotoxicity in 5–8 F and HNE2 than HK1 and C666-1 cells (Fig. 5l). These findings suggest that the ACTBL2/UNC13D-based molecular classification may serve as a potential biomarker for guiding personalized drug selection in NPC.

### Panobinostat inhibits homologous recombination by targeting MYC in NPC

**Fig. 6:**
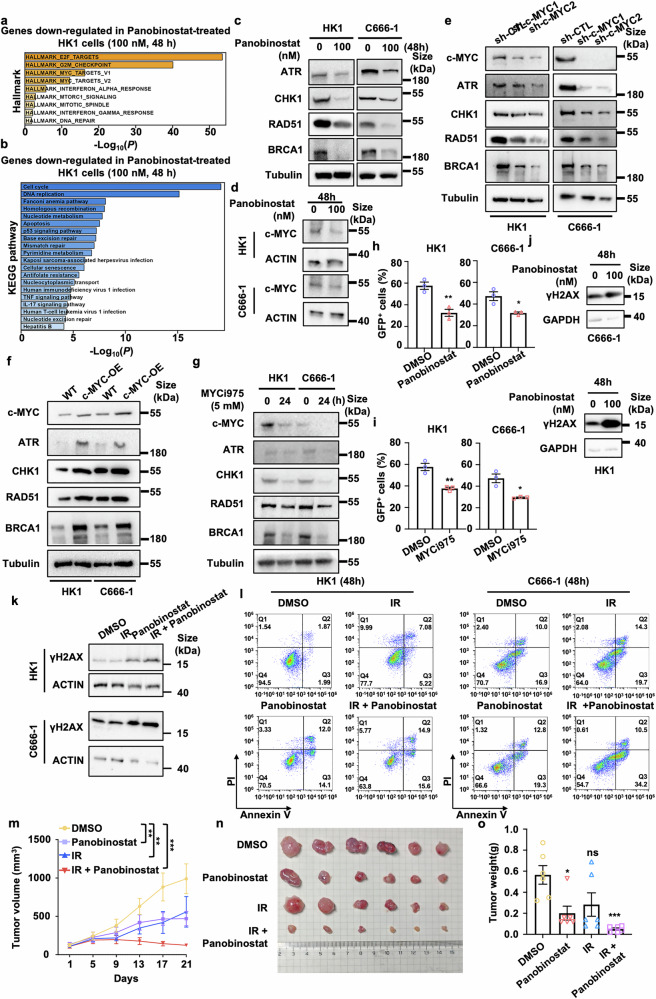
Panobinostat inhibits HR DNA repair through suppressing MYC-mediated gene transcriptional activation, and combination with IR exhibits synergistic effects. **a**, **b** HK1 cells treated with DMSO or Panobinostat (100 nM) for 48 h were subjected to RNA-seq analysis. Enriched Hallmark gene sets (**a**) and KEGG pathways (**b**) for downregulated genes in Panobinostat-treated cells are shown. **c**, **d** HK1 and C666-1 cells were treated with DMSO or Panobinostat (100 nM) for 48 h, followed by immunoblotting (IB) analysis to examine the expression of proteins as indicated. **e** HK1 and C666-1 cells were infected with lentivirus expressing control shRNA (shCTL) or tow individual shRNAs targeting c-MYC (sh-c-MYC1 or sh-c-MYC2) for 72 h, followed by IB analysis to examine the expression of proteins as indicated. **f** HK1 and C666-1 cells were transfected with control vector or vector expressing c-MYC (c-MYC-OE) for 72 h, followed by IB analysis to examine the expression of proteins as indicated. **g** HK1 and C666-1 cells were treated with or without MYCi975 (5 μM) for 24 h, followed by IB analysis to examine the expression of proteins as indicated. **h**, **i** HK1 and C666-1 cells were first transfected with the DR-GFP plasmid for 72 h before transfecting with I-Sce plasmid for 24 h. Cells were treated with or without Panobinostat (**h**) or MYCi975 (**i**) for 48 h before washing with ice-cold PBS and then subjected to flow cytometry analysis to quantify the proportion of GFP-positive cells. **j** HK1 and C666-1 cells treated with DMSO or Panobinostat (100 nM) for 48 h were subjected to IB analysis with antibodies as indicated. **k** HK1 and C666-1 cells were treated with Panobinostat (50 nM, 48 h) or IR (6 Gy, 12 h) alone or in combination, followed by IB analysis with antibodies as indicated. **l** Cells as described in (**k**) were stained with Annexin V-FITC and PI, followed by flow cytometry analysis. **m** Female BALB/c nude mice (4 weeks-old) were subcutaneously inoculated with C666-1 cells (5 × 10⁶ cells/mouse) in the right flank. When tumors reached 50 mm³ in volume, tumor-bearing mice were randomized into control, monotherapy, and combination therapy groups (*n* = 6 per group). Treatments consisted of Panobinostat monotherapy (5 mg/kg, intraperitoneal injection every three days for three weeks), localized radiotherapy (8 Gy single-dose irradiation), or their combination (Radiotherapy was administered 24 h prior to the first Panobinostat dose). The growth curves of tumors are shown (mean ± SEM; *P < 0.05; **P < 0.01; ***P < 0.001). *P* values were calculated by two-way ANOVA. **n** Mice as described in (**m**) were sacrificed, and tumors were collected and imaged. **o** The weight of tumors as shown in (**n**). (mean ± SEM; * *P* < 0.05; ***P < 0.001; ns non-significant)

Our experimental results demonstrated that Panobinostat significantly inhibits NPC progression in both cellular and mouse tumor models. We next explore the underlying mechanisms by which Panobinostat exerts its anti-tumor function in NPC. HDACs are a family of enzymes that play pivotal roles in chromatin structure modification and gene expression regulation.^54^ Panobinostat exhibits potent inhibitory activity at low nanomolar concentrations against all Class I, II, and IV purified recombinant HDAC enzymes, exerting its anti-tumor effects primarily through epigenetic regulation of gene expression and inhibition of protein metabolism. To further investigate the mechanisms, we performed RNA sequencing (RNA-seq) on control and Panobinostat-treated HK1 cells. Hallmark enrichment analysis revealed that Panobinostat treatment significantly downregulated E2F-targets, G2/M checkpoint regulators, and MYC-associated oncogenic pathways, among others, in HK1 cells (Fig. 6a and Data S8). KEGG pathway analysis confirmed Panobinostat-mediated inhibition of cell cycle, DNA replication, and homologous recombination (HR) repair mechanisms, among others (Fig. 6b and Data S8).

DNA damage triggers replication fork stalling, leading to the accumulation of repair proteins at these sites. This process activates the ataxia telangiectasia and Rad3-related (ATR) protein kinase, which phosphorylates and activates checkpoint kinase 1 (CHK1), inducing cell cycle arrest to facilitate DNA repair. ATR also promotes HR repair by activating RAD51 through CHK1-mediated phosphorylation of BRCA1. In our RNA-seq analysis, Panobinostat-treated HK1 cells exhibited marked suppression of ATR, CHEK1, RAD51, and BRCA1 expression compared to control cells, which was validated at both mRNA and protein levels in HK1 and C666-1 cells (Fig. 6c, Fig. S4a and Data S8). Considering that the cell cycle affects the expression of HR pathway-related proteins, which are often highly expressed in the S/G2 phase.^55^ To determine whether Panobinostat affects the expression levels of HR pathway proteins is due to cell cycle regulation, we examined the cell cycle profile of NPC cells treated with Panobinostat. The results showed that HK1 and C666-1 were arrested in the S/G2 phase in response to Panobinostat treatment (Figs. S4b and S4c). Therefore, the downregulation of HR pathway molecules by Panobinostat is not due to its effects on cell cycle regulation. Previous studies suggests that c-MYC modulates HR-related gene expression.^56^ Therefore, we quantified c-MYC expression following Panobinostat treatment and observed substantial reductions in c-MYC mRNA and protein levels in both HK1 and C666-1 cells (Fig. 6d and Fig. S4a). We then tested whether MYC regulates the expression of HR-related genes, including ATR, CHEK1, RAD51, and BRCA1. Knockdown or overexpression of c-MYC in both HK1 and C666-1 cells resulted in reciprocal changes in the mRNA and protein levels of ATR, CHK1, RAD51, and BRCA1 (Fig. 6e, f, and Figs. S4d and S4e). Additionally, the c-MYC inhibitor MYCi975 reduced the mRNA and protein levels of ATR, CHK1, RAD51, and BRCA1 (Fig. 6g and Fig. S4f). Using a GFP reporter-based HR detection system followed by flow cytometry analysis, we found that Panobinostat reduced HR repair efficiency in both HK1 and C666-1 cells (Fig. 6h), with similar effects observed with MYCi975 (Fig. 6i). Deficiency in HR repair leads to DNA double-strand breaks (DSBs), and γH2AX serves as a well-established biomarker for DSBs.^57,58^ Accordingly, we detected elevated γH2AX levels in Panobinostat-treated cells through both immunoblotting and immunofluorescence analysis (Fig. 6j and Fig. S4g). This observation aligns with previous reports demonstrating Panobinostat-induced genomic instability in cancer cells.^59^ Given the established link between DNA damage and apoptosis, Panobinostat significantly induced apoptosis (Fig. 5d, e). These findings suggest that Panobinostat impairs HR repair capacity by suppressing the expression of c-MYC as well as its downstream targets including ATR, CHK1, RAD51, and BRCA1. By inhibiting critical components of the DNA damage response/HR repair pathway, Panobinostat exacerbates DSBs accumulation and enhances apoptosis in NPC cells.

Panobinostat promoted DNA damage accumulation by inhibiting HR repair, a phenotype that may sensitize tumors to radiotherapy. When combined with ionizing radiation (IR), Panobinostat significantly increased γH2AX levels and induced apoptosis in HK1 and C666-1 cells compared to monotherapy (Fig. 6k, l, and Figs. S4h, S4i). Similarly, in a subcutaneous xenograft mouse model, the combination of Panobinostat and IR synergistically inhibited tumor growth compared to either treatment alone, as evidenced by significantly reduced tumor volume and weight (Fig. 6m–o), with no impact on body weight (Fig. S4j). These findings establish a mechanistic link between HR DNA damage repair and Panobinostat’s radiosensitizing effects in NPC.

## Discussion

Our study integrated large-scale proteomic and phosphoproteomic datasets from clinical samples, providing critical insights into NPC biology. While genomic studies of NPC patients in Chinese cohorts have identified some recurrent mutations and oncogenic drivers, proteomic profiling goes further by revealing translational and post-translational dysregulation, which complement genomic findings.

Our proteomic analysis systematically revealed multi-layered molecular alterations in NPC. We observed widespread dysregulation of cell cycle control, involving established regulators such as PBK,^60^ CDK1,^61^ KPNA2,^62^ and so on, alongside novel effector proteins like TK1 and GINS4 that have not been previously studied in this cancer. Notably, the expression of NME1,^63^ traditionally regarded as a tumor suppressor, showed an upward trend in our cohort, which contrasts with prior understanding. Concurrently, cilia-associated proteins were significantly downregulated, consistent with prior RNA-seq data.^64,65^ Low SPAG6^66,67^ expression correlated with poor prognosis in NPC, warranting functional validation. Both SEPTIN7,^68^ a known tumor suppressor, and SEPTIN6,^69–71^ which exhibits context-dependent roles in cancer, were markedly downregulated. Their reduced expression strongly predicted adverse outcomes, suggesting tumor-inhibitory functions in NPC and highlighting the need to investigate their methylation-driven silencing.^72^ At the metabolic level, several regulators including ADH1B, ADH1C, and GSTA2 were downregulated. Higher expression of ADH1C correlated with more favorable patient survival, whereas low GSTA2 expression was linked to a poorly differentiated tumor state, highlighting the context-dependent nature of its function.^73^ Further analysis revealed significant upregulation of metastasis-associated molecules such as FERMT1, ITGB6, and members of the PLOD family. Their elevated expression was closely associated with shortened patient survival, and these molecules likely promote metastatic progression by mediating collagen modification and microenvironment remodeling.^74^ Furthermore, the mTOR signaling pathway was significantly activated in NPC tissues. Key components like P4HA1^75^ and SERPINH1^76^ have been integrated into metastasis and cell cycle-related pathways, collectively driving tumor progression. Proteomic profiling revealed significant immune dysregulation in NPC. Interferon-stimulated genes, such as ITGB6 and KPNA2, were markedly upregulated—a pattern linked to EBV persistence—and their enrichment was associated with poor prognosis. Although complement receptors CR1/CR2^77,78^ were downregulated in tumors, their high expression paradoxically correlated with worse outcomes, suggesting an immunosuppressive microenvironment. B‑cell markers were suppressed, indicating impaired immunity. HLA‑DOB overexpression predicted poor prognosis, contrasting with favorable outcomes associated with high HLA‑DRA/DQB1/DRB3. Non‑canonical regulators like ADRA2A and CD9 were also identified. Furthermore, the phosphoproteomic signature of NPC, characterized by significant hyperphosphorylation of cell cycle regulatory proteins, shows conserved patterns across multiple malignancies.^14^ Kinase activity prediction analysis further highlighted marked activation of CDK1, CDK2, and AURKA, which are involved not only in cell cycle progression but also in mediating radioresistance via DNA damage repair and cell survival pathways.^57^ The observed upregulation of these kinases provides a mechanistic rationale for combining CDK/AURKA inhibitors with radiotherapy to enhance tumor cell killing, particularly in radioresistant NPC.

In this study, we performed proteomic stratification of NPC and identified two molecular subtypes with distinct clinical behaviors. The S1 subtype, associated with better outcomes, showed enhanced proliferative activity marked by upregulation of cell cycle and oncogenic pathways. In contrast, the S2 subtype, linked to poorer prognosis, exhibited a proteomic signature dominated by activation of coagulation and complement signaling. Based on prior literature, we speculate that this hypercoagulable phenotype may promote immunothrombosis-mediated immune evasion, while complement activation could further suppress antitumor T-cell responses,^79^ together fostering an immunosuppressive niche that likely drives S2 progression. NPC remains therapeutically challenging due to a lack of effective targeted therapies, but our proteomic analysis identified HDAC inhibitors as dominant predictions. The pan‑HDAC inhibitor Panobinostat showed superior antitumor efficacy in cellular and animal models. RNA sequencing revealed that Panobinostat significantly suppressed MYC targets, particularly key homologous recombination repair molecules. Intriguingly, although an HDAC inhibitor typically enhances transcription, Panobinostat induced transcriptional downregulation of repair factors—a phenomenon consistent with reported mechanisms^80^ in which pan‑HDAC inhibition disrupts super‑enhancer architecture and downregulates master regulators such as c‑MYC, providing a molecular rationale for the observed transcriptional modulation. In the immunosuppressive microenvironment, whether Panobinostat can remodel the immunosuppressive NPC microenvironment remains an open question. For the S2 subtype of NPC, we computationally predicted potential therapeutic agents, including anti-angiogenic drugs, anti-metastatic drugs, and immunomodulatory drugs. Anti-angiogenic agents target S2’s dense vascular network dependence by inhibiting vascular endothelial cell proliferation/migration and downregulating pro-angiogenic factors. Anti-metastatic drugs are prioritized, as they disrupt tumor cell-extracellular matrix interactions via MMP regulation to counter S2’s complement-mediated MMP-driven metastasis. For immunomodulatory agents, the potential inhibition of S2-subtype tumors may be attributed to their immunosuppressive characteristics. Targeting the inflammatory state of this subtype could suppress tumor growth—an inference based solely on features of the immune microenvironment, the underlying mechanisms of which require further experimental clarification. These underexplored oncology drugs provide a preliminary research direction for the S2 subtype.

Although this study has achieved preliminary progress in molecular subtyping, biomarker identification, and targeted therapy exploration for NPC, several limitations remain that require further refinement. First, all samples were obtained from nasopharyngeal biopsy tissues, which are limited in size and make it difficult to accurately separate tumor cells from stromal components. This may affect the accuracy of subtype characterization. Additionally, the initial training set used to construct the diagnostic model for NPC and its subtypes was limited in sample size. Although preliminary validation was performed using external data, the generalizability of the associated biomarkers still requires further confirmation through larger-scale, multi-center independent clinical cohorts. Second, in validating the efficacy of targeted drugs, only two cell lines per subtype were used for in vitro experiments, limiting the depth of analysis. Future studies should employ patient-derived xenograft (PDX) models and immunocompetent humanized mouse models for more comprehensive in vivo efficacy evaluation. Finally, although the combination of Panobinostat and radiotherapy has shown preliminary feasibility in animal models for treating NPC, its clinical translational value needs to be further substantiated through follow-up clinical trials.

In summary, by integrating large-scale proteomic and phosphoproteomic datasets, this study systematically reveals key molecular dysregulations in NPC, including widespread aberrations in cell cycle control, metabolism, metastasis, immune signaling, and kinase pathways. Proteome-based subtyping (S1 and S2) provides a new basis for prognosis prediction and individualized treatment. The HDAC inhibitor Panobinostat exhibits potent antitumor activity, with a mechanism involving the suppression of MYC and downstream homologous recombination repair factors, supporting its combination with radiotherapy. Although limitations remain in model validation, the proteomic landscape and potential therapeutic strategies established here lay an important foundation for precision medicine in NPC.

## Materials and methods

### Clinical sample acquisition

The studies involving human participants were reviewed and approved by Clinical Research Ethics Committee of the First Affiliated Hospital of Xiamen University (approval No. 2024-108). All the patients presented with informed consent preoperatively and received no treatment prior to biopsy. All biopsy tissues were obtained via nasopharyngeal endoscopy. NPC tissues were collected from patients diagnosed with NPC, while normal control tissues were obtained from individuals suspected of having NPC but ultimately confirmed to be negative. Altogether, we collected the primary tumors of 87 NPC patients, as well as 56 non-cancerous nasopharyngeal samples of normal subjects from the First Affiliated Hospital of Xiamen University (Data S1). Another clinical cohort included tissue microarrays from 89 NPC patients from SHANGHAI OUTDO BIOTECH CO., LTD. (Data S6). All cases were classified according to the 8th edition of the American Joint Committee on Cancer (AJCC) pTNM system. Clinical information regarding smoking, alcohol consumption, and histopathological factors was obtained from medical records. Overall survival (OS) was defined as the interval from diagnosis to death or from diagnosis to the last observation for surviving patients. Progression-free survival (PFS) was defined as the interval from diagnosis to the confirmation of recurrence, metastasis, or death.

### Cell lines, organoids, and reagents

HK1 (EBV^-^) and C666-1 (EBV^+^) cells were cultured in RPMI-1640 medium supplemented with 10% fetal bovine serum (FBS) and 1% Penicillin /Streptomycin (P/S), while 5–8 F (EBV^-^) and HNE2 (EBV^-^) cells were cultured in DMEM supplemented with 10% FBS and 1% P/S. All cells were maintained under the humidified 5% CO_2_ atmosphere at 37 °C. One strain of NPC organoids was purchased from Xiamen Mogengel. The cryopreserved organoid vials stored in liquid nitrogen were retrieved and rapidly thawed in a 37 °C water bath for 1–2 min. After thawing, 5 volumes of organoid-specific medium (MB-0818L07, Xiamen Mogengel) were added, followed by gentle pipetting to mix thoroughly. The mixture was centrifuged at 1000 rpm for 5 min, and the supernatant was discarded. The organoid pellet was resuspended in complete medium containing Matrigel, which was then dropped into a culture dish to form microdroplets. The microdroplets were incubated in a 37 °C, 5% CO₂ incubator for 20 min to allow Matrigel solidification. After solidification, sufficient organoid medium was added, and the medium was refreshed every 2–3 days. The organoids were continuously cultured for 3–5 days until their morphology stabilized. Droxinostat (S1422), KU-0063794 (S1226), and Panobinostat (S1030) were purchased from Selleck Chemicals. Treprostinil (HY-100441), Evodiamine (HY-N0114), Griseofulvin (HY-17583), and GW-843682 (HY-11003) were purchased from MedChemExpress. Emetine (M2666) was purchased from Abmole.

### Plasmids

Lentiviral expression vector of MYC was purchased from PPL (Public Protein/Plasmid Library). ShRNA targeting MYC were cloned into pPLK vector (targeting sequence: 5’-CAGTTGAAACACAAACTTGAA-3’ and 5’-CAATGACACCCTGTACTTCTT-3’). The plasmids pDRGFP and pCBASceI were purchased from Addgene.

### Animals

Female BALB/c nude mice (age 4–6 weeks) were purchased from Charles River Laboratories Company (Beijing, China). All animal experiments were conducted following a protocol approved by the Institutional Animal Care and Use Committee of the Xiamen University Laboratory Animal Center. Mice were maintained in animal room with 12 h-light/12 h-dark cycles at Animal Facility in Xiamen University. For tumor implantation, C666-1 cells (5 × 10^6 cells per mouse) were suspended in RPMI-1640 medium without FBS and subcutaneously inoculated into the right hind limb of each mouse. Mice were then blindly randomized into experimental groups (*n* = 6 per group). Two sets of treatment regimens were implemented in the experiment. One set of treatments included intraperitoneal administration of either vehicle control (DMSO) or therapeutic agents (Panobinostat, 10 mg/kg; KU-0063794, 3 mg/kg; Droxinostat, 15 mg/kg) three times per week for four consecutive weeks, continuing until the predefined experimental endpoint. The other set of treatments consisted of Panobinostat monotherapy (5 mg/kg, intraperitoneal injection every three days for three weeks), localized radiotherapy (8 Gy single-dose irradiation), or their combination (Radiotherapy was administered 24 h prior to the first Panobinostat dose). The length and width of the tumors (in millimeters) were measured 3 times every week with calipers. Tumor volume was calculated using the formula (A*B^2^)/2, where A and B were the long and short dimensions, respectively. Mice were blindly randomized into different groups for treatment studies.

### LC-MS/MS analysis

#### Protein extraction and digestion

The NPC and normal nasopharyngeal tissue samples were processed according to the Filter Aided Sample Preparation (FASP) method as described previously.^15^ Briefly, the tryptic peptides were desalted using StageTips and then lyophilized. Subsequently, they were labeled with TMTpro 16plex (Pierce) as per the manufacturer’s instructions. All samples were taken in equal amounts to mix for the “internal reference” used in TMT labeling. For each set of TMT labeling experiment, 100 µg of peptides from mixed samples per EP tube were used as internal reference. 1.6 mg labeled peptides were off-line fractionated by bRP using a Waters XBridge BEH C18 5 μm 4.6 × 250 mm column on an Ultimate 3000 high-pressure liquid chromatography (HPLC) system (Dionex) operating at 1 mL/min. Peptides were separated by a linear gradient from 5 to 40% B (5 mM ammonium formate, 90% ACN) in 66 min followed by a linear increase to 70% B in 6 min. A total of 72 peptide fractions were collected and sequentially numbered. These 72 fractions were first concatenated into 24 fractions (i.e., fractions 1, 25, 49 were combined; fractions 2, 26, 50 were combined, and so forth). For global proteome analysis, 5% of each of these 24 pooled fractions was aliquoted and reserved. The remaining 95% portion of the 24 fractions was renumbered, then further concatenated into 8 fractions (i.e., fractions 1, 9, 17 were combined; fractions 2, 10, 18 were combined, etc.). These 8 fractions were subjected to phosphopeptide enrichment using the Fe-NTA kit (Thermo Scientific, A32992) in strict accordance with the manufacturer’s instructions. Finally, all prepared peptide samples (both for proteome and phosphoproteome profiling) were lyophilized prior to LC-MS/MS analysis.

#### TMTpro 16-plex labeling

The 87 NPC tumor and 56 normal nasopharyngeal tissue samples were labeled in 10 groups of TMTpro 16plex experiments for LC-MS/MS analysis. For each TMTpro 16plex experiment, the mixed peptides were labeled with channel 131 C as the internal reference, and all NPC tumor and normal nasopharyngeal tissue samples were labeled with the other 15 channels (Tumor nasopharyngeal tissues labeled with 127 N, 128 N, 129 N, 130 N, 131 N, 132 N,133 N, and 134 N; non-tumor nasopharyngeal tissues labeled with 126, 127 C, 128 C, 129 C, 130 C, 132 C, and 133 C).

#### Proteomic and phosphoproteomic LC-MS/MS analysis

MS experiments were performed on Orbitrap Fusion Lumos (Thermo Fisher Scientific). The peptides were dissolved in 0.1% formic acid (FA) and separated on an analytical column (75 μm × 25 cm) packed with 2 μm C18 beads (Thermo Fisher Scientific) using a linear gradient ranging from 9 to 40% B (80% ACN and 0.1% FA) in 100 min and followed by a linear increase to 50% B in 20 min at a flow rate of 300 nL/min. The MS was operated in data-dependent acquisition (DDA) mode. The spray voltage was set at 2.2 kV and the temperature of ion transfer capillary was 300 °C. The MS spectra (350–1500 m/z) were collected with 60,000 resolution, AGC of 4 × 105 and 50 ms maximal injection time. Selected ions were sequentially fragmented by HCD with 38% normalized collision energy in a 3 s cycle, specified isolated windows 0.7 m/z, 50,000 resolution. AGC of 1 × 105 and 105 ms maximal injection time were used. Dynamic exclusion was set to 30 s.

#### MS data analysis

The data were collected using Xcalibur software (Thermo Fisher Scientific, version 3.0). Raw data was processed using Proteome Discoverer (PD, version 2.4), and MS/MS spectra were searched against the Uniprot human proteome database. Search parameters were as follows: 20 ppm tolerance of precursor mass error, 0.02 Da tolerance of fragment mass error, variable modification for oxidation (Met) ( + 15.9949 Da), TMTpro (Lys) (304.207 Da), phosphorylation (Ser, Thr, Tyr) ( + 79.966 Da) and acetylation (protein N-terminus) ( + 42.0106 Da), carbamidomethylation (Cys) ( + 57.0215 Da), TMTpro (N-terminal) (304.207 Da) as fixed modification. The peptide and protein identifications were filtered by PD to control the false discovery rate (FDR) < 1%. At least one unique peptide was required for protein identification.

### Proteomic data analysis

#### Data normalization

The protein expression ratio is the ratio of sample abundance to “internal reference” mixed sample abundance. To reduce sample-specific bias in protein level quantification, expression ratios are log2-transformed and normalized using mean centering across all proteins. In normalized samples, proteins should have a log2-transformed expression ratio centered at zero.

#### Data filtering

The proteomic data was filtered to five datasets at different levels as the following criteria. Dataset 1 (Prot1): all proteins quantified in at least one of 10 TMT groups. Dataset 2 (Prot2): proteins quantified with high confidence in at least one of 10 TMT groups. Dataset 3 (Prot3): proteins quantified with high confidence in at least half samples. Dataset 4 (Prot4): proteins quantified in all samples. Dataset 5 (Prot5): proteins quantified with high confidence in all samples.

#### Batch effect analysis

The unsupervised PCA and hierarchical clustering were performed on protein expression ratios in Prot5 to assess batch effect due to TMT multiplexes in R v.4.4.1 based on group (Tumor/non-tumor) and run-id (Batch 1–10). For PCA, used R package base for confidence intervals. For hierarchical clustering, used complete linkage with Euclidean distance by R package pheatmap v.1.0.12.

#### Differential expression analysis between tumor and non-tumor

Differential expression analysis between tumor and non-tumor samples was performed on proteins in Prot1. The statistical significance was calculated by two-sided wilcoxon rank-sum test. Proteins with BH adjusted *P* value < 0.01 and FC (Fold change, ratio of average protein expression ratio between tumor and non-tumor samples) > 1.2 or < 0.83 were considered to be significantly upregulated or downregulated in tumor samples.

#### Differential expression analysis between EBV-positive and EBV-negative NPC

Differential expression analysis was performed on proteins in Prot1 between EBV-positive and EBV-negative NPC. The EBV infection status was shown in Data S1. The statistical significance was calculated by two-sided wilcoxon rank-sum test. Proteins with *P* value < 0.05 and FC (Fold change, ratio of protein expression between tumor and non-tumor samples) > 1.2 or < 0.83 were considered to be significantly upregulated or downregulated in tumor samples, respectively.

#### Correlation between protein expression ratios and clinical outcome

We evaluated the association between differential expression protein expression ratios and patient risk by Cox PH model. A univariate Cox PH model was used to estimate the hazard ratio (HR), confidence interval, and Cox *P* value of each protein. HR > 1 means that the expression of the protein is positively correlated with patient risk, while HR < 1 means a negative correlation. The correlation is considered significant if log-rank *P* value < 0.05. Correlation with OS and DFS are estimated separately.

### Phosphoproteomic data analysis

#### Quantification and normalization of phosphosites

Each phosphopeptide’s abundance is determined by the sum of the three final eluted fractions. Phosphosite abundance is determined by the median abundance of all phosphopeptides matching that site. The expression abundance of the phosphosites was subjected to quantile normalization implemented in the R package limma v.3.42.2. Missing values were imputed with the minimum value in the phosphoproteomic data.

#### Phosphoproteomic data filtering

The phosphoproteomic data was filtered to three datasets at different levels using the following criteria. Dataset 1 (Phos1): Phospho-sites quantifiable in at least one of the 10 groups. Dataset 2: Phospho-sites quantifiable in at least half sample. Dataset 3: Phospho-sites quantifiable in all samples.

#### Differential phosphoproteomic analysis between tumor and non-tumor samples

Differential phosphoproteomic analysis between tumor and non-tumor samples was performed on phosphosites in Phos1. The statistical significance was calculated by two-sided wilcoxon rank-sum test. Phosphosites with BH adjusted *P* value < 0.01 and FC (Fold change, ratio of average phosphosite abundance between tumor and non-tumor samples) > 1.5 or < 0.67 were considered to be significantly upregulated or downregulated proteins in tumor samples.

### Proteomic subtyping analysis

#### Consensus clustering for proteomic data

The consensus clustering method was used to implement the consensus clustering method to identify subtypes of NPC by R package ConsensusClusterPlus. The top 5,000 genes with the largest changes based on MAD (median absolute deviation) was selected for clustering analysis. The main settings are as follows: maximum cluster number (maxK) = 6, number of repeats (reps) = 1000, proportion of items to sample (pItem) = 0.8, proportion of features to sample (pFeature) = 0.8, cluster algorithm (clusterAlg) = “hc” (hierarchical clustering), distance = “pearson” and seed = 314. The average silhouette width which determine the optimal number of clustering was calculated using the R package cluster v.2.1.0.

#### Prognostic assessment of molecular subtypes

Kaplan–Meier curves was used to evaluate the OS and PFS difference between the two molecular subtypes, S1 and S2. The *P* value was calculated by log-rank tests.

#### The differential expression analysis between the two molecular subtypes

Differential expression analysis between the two molecular subtypes was performed on proteins in Prot1. The statistical significance was calculated by two-sided wilcoxon rank-sum test. Proteins with BH adjusted *P* value < 0.01 and FC (Fold change, ratio of average protein expression ratio between S2 and S1 subtype) > 1.2 or < 0.83 were considered to be significantly upregulated or downregulated in molecular subtypes samples.

#### Estimation of STromal and Immune cells in MAlignant Tumor tissues using Expression data (ESTIMATE) analysis for molecular subtypes

Immune cell and stromal cell score for all samples were calculated by the R package estimate.

#### Immunoinfiltration analysis for two molecular subtypes

Single-sample gene set enrichment analysis (ssGSEA) was performed using R package GSVA (version 1.46.0)^81^ to calculate the ssGSEA scores for immune cell gene set. The gene sets used in the ssGSEA analysis were retrieved from the following published study.^82^

### Functional enrichment analysis

The differentially expressed proteins and phosphoproteins were used to infer enriched KEGG pathways, Hallmark gene sets, and GO biological functions by R package clusterProfiler. The differentially expressed proteins and phosphoproteins included: (1) upregulated or downregulated proteins in tumor vs. non-tumor samples; (2) upregulated or downregulated phosphoproteins in tumor vs. non-tumor samples; (3) upregulated or downregulated proteins in molecular subtypes vs. non-tumor samples; (4) upregulated or downregulated phosphoproteins in molecular subtypes vs. non-tumor samples.

### Tumor diagnostic model

#### Signature identification

The differential proteins in tumor vs. normal-tumor samples were used as the initial feature set for feature identification. Feature selected was implemented on the initial feature set by the R package mlr and the maximum number of features was limited to no more than 3. Support vector machine (SVM) was chosen as the classifier because it has good generalization ability. Parameters were set as follows: feature selection method = “sfs” (sequential forward search), resampling algorithm = “Subsample”, number of resampling = 50, and performance measures = “auc”. We set the maximum number of features (“max.features”) to 1, 2 or 3, and repeated the feature selection process 100 times. Feature combinations that are frequently identified during feature selection are considered as robust signatures.

#### Signature evaluation

The classification performance of each signature was evaluated using five-fold cross-validation. For each cross-validation fold, the dataset was randomly partitioned into five equally sized subsets. Four of these subsets were used to train a Support Vector Machine (SVM) model for each signature, while the remaining subset served as the test set. This process was iterated five times, ensuring that each subset served as the test set exactly once. The performance of the SVM model for each signature was then measured by its Area Under the Receiver Operating Characteristic Curve (AUC) and accuracy on the held-out test set. To ensure an unbiased and robust evaluation, this entire five-fold cross-validation procedure was repeated 100 times, and the results were averaged.

#### Subtype prediction

For the 89 NPC patient samples included in the tissue microarray, the H-Scores of ACTBL2 and UNC13D were first subjected to log₂ transformation and further normalized via Z-score transformation. Subsequently, the normalized H-Scores of ACTBL2 and UNC13D for each patient were input into the subtype diagnostic model to predict the probability of their classification into the S1 or S2 subtype.

### CMAP-based drug prediction

We used CAMP to predict potential drugs for NPC and two Subtypes. We first calculated the significance ranking by −10 * log(FC) * (*P* value), setting the top 150 most significantly changed proteins as signatures. The signatures were then compared to each reference gene-expression profile. When upregulated proteins tended to appear near the top of the list and down-regulated proteins near the bottom, it was named “positive connectivity”. Conversely, it was named “negative connectivity”, yielding a “connectivity score” ranging from +1 to −1. The connectivity score of each perturbagen was calculated using the signatures. We sorted perturbagens according to their connectivity scores in increasing order. And the top drugs with the highest negative connectivity scores were predicted as potential drugs.

### Plasmids transfection, lenti-viral vectors packaging and infection

Plasmids transfection was carried out by employing Lipofectamine 2000 (Life Technology) or Transfect EZ 3000 Plus (Zhong Ke Xin Chuang Biotech) in accordance with the manufacturer’s protocol. Briefly, HEK293T cells were seeded in the culture plates coated with poly-D-lysine (0.1% (w/v), Sigma, P7280) and transfected with the lenti-viral vector along with the packaging vectors, pMDL, VSVG, and REV, at a ratio of 10:5:3:2 using Transfect EZ 3000 Plus. After 48 h, the virus was collected, filtered, and added to HK1 and C666-1 cells in the presence of 10 mg/mL polybrene (Sigma, H9268), followed by centrifugation for 30 min at 1500 × *g* at 37 °C. The medium was replaced 24 h later.

### RNA Isolation and real-time quantitative PCR (RT-qPCR)

Total RNA was isolated using Trizol (Invitrogen) following the manufacturer’s protocol. First-strand cDNA synthesis from total RNA was carried out using GoScript™ Reverse Transcription Mix (Promega, random primers), followed by quantitative PCR (qPCR) using AriaMx Real-Time PCR machine (Agilent Technologies). qPCR was performed using AriaMx Real-Time PCR machine (Agilent Technologies). All RT-qPCRs were repeated at least three times, and the relative abundance of each transcript was normalized to the expression level of GAPDH. Sequence information for all the primers used were presented in Supplementary Data 9.

### RNA Sequencing (RNA-seq)

To prepare RNA for sequencing, total RNA was isolated using RNeasy Mini Kit (Qiagen) following the manufacturer’s protocol. DNase I in column digestion was included to ensure the RNA quality. RNA library preparation was performed by using NEBNext® UltraTM Directional RNA Library Prep Kit for Illumina(E7420L). Paired-end sequencing was performed with Illumina HiSeq platform at Amogene Biotech Co., Ltd.

BCL files were demultiplexed and converted to fastq files by using bcl2fastq (version 2.20), and then fastp (version 0.19.10) was used to trim adapter and filter out low quality reads. Sequencing reads were aligned to hg19 reference genome by using Tophat^83^(http://ccb.jhu.edu/software/tophat/index.shtml). Cuffdiff was used to quantify the expression of RefSeq annotated genes with the option -M (reads aligned to repetitive regions were masked) and -u (multiple aligned reads are corrected using ‘rescue method’).^83^ Coding genes with FPKM (fragments per kilobase per million mapped reads) larger than 0.5 in any of the experimental conditions were included in our analysis. FPKM of a gene was calculated as mapped reads on exons divided by exonic length and the total number of mapped reads.

### Western blotting

Proteins were extracted from cells using RIPA Lysis Buffer (E-BC-R327, Elabscience). The protein lysates were quantified by BCA assay (23250, Thermo Fisher). Samples were then loaded onto SDS-PAGE gels and electrophoresed at a constant voltage. Subsequently, proteins were transferred to a PVDF using wet transfer system. The membrane was blocked with 5% skim milk in TBST (50 mM Tris-HCl (pH 7.4), 150 mM NaCl, 0.1% Tween-20) for 1 to 2 h at room temperature. Next, primary antibodies specific to the target proteins were added and incubated overnight at 4 °C. After washing with TBST for 5 times, the membrane was incubated with the appropriate secondary antibody conjugated to horseradish peroxidase (HRP) for 1 h at room temperature. Finally, the protein bands were visualized using an enhanced chemiluminescence (ECL) detection system, and the images were captured using a gel imaging system. Rabbit anti-ATR polyclonal antibody (A21253, Abclonal), rabbit anti-CHEK1 antibody (A4194, Abclonal), rabbit anti-RAD51 antibody (A6268, Abclonal), rabbit anti-BRCA1 antibody (22362-1-AP, Proteintech), rabbit anti-cMYC antibody (67447-1-IG, Proteintech) and rabbit anti-ACTBL2 antibody (DF4799, Affinity Biosciences) were diluted at 1:1000. Mouse anti-α-Tubulin monoclonal antibody (A6830, Abclonal) and mouse anti-UNC13D monoclonal antibody (67193-1-Ig, Proteintech) was diluted at 1:5000.

### Cell proliferation assay

The cells were digested and cultured in a medium containing 10% serum to form a single cell suspension. The cells were counted and inoculated into 96-well plates with 1,000 cells per well (100 μL/well). The cells were cultured for 12 h to adhere to the cell well, and then the culture medium with different drug concentration was changed. After incubation for different duration, 10 μL CCK8 (GK10001, GlpBio) was added into each well for 1 h. The absorbance value of each well was measured at the wavelength of 450 nm using Infinite F50 Plus microplate reader (TECAN). The cell viability at different drug concentrations was calculated and then mapped with GraphPad prism 9 software.

### Colony formation assay

In the 6-well plates, 500–1000 cells per well were inoculated. After 24 h, the cells adhered to the well. Different concentrations of drugs were added to the cell culture medium, and the culture medium was replaced with drug every two days for 2 weeks. After washing with cold PBS, cells were fixed with cold 4% paraformaldehyde for 20 min and stained with crystal violet for 15 min. Each experiment was done in triplicate.

### ATP detection in organoids

Organoids with uniform morphology and good viability were selected and transferred to a 96-well plate (approximately 50 organoids per well). After culturing for 3–5 days until the organoid morphology stabilized, different drugs were added for treatment, followed by incubation at 37 °C with 5% CO₂ for 72 h. After incubation, the 96-well plate was placed under an inverted microscope, and 3–5 random fields of view were selected for each group to observe and record the size, integrity, and structural changes of organoids. Imaging was performed with a Leica DMi8 microscope. After photography, the medium in the wells was aspirated and discarded, and 100 μL of ATP detection reagent diluted according to the kit instructions (Beyotime, C0056L) was added to each well, followed by shaking incubation at room temperature in the dark for 10 min. Subsequently, the supernatant in each well was transferred to a microplate reader (SpectraMax i3x) to detect the fluorescence intensity with the fluorescence value indirectly reflecting ATP content and cell viability. Finally, the relative viability of each drug group was calculated with the control group viability set as 100%, and statistical analysis was performed using Prism 9 software.

### Cell cycle by flow cytometry

The cells treated with 100 nM panobinostat or DMSO for 72 h were collected and rinsed with cold PBS. Subsequently, the cells were incubated with RedNucleus I (C6078S, UElandy) in binding buffer for 20 min in the dark. The samples were analyzed on a flow cytometer, and the proportions of cells in G0/G1, S, and G2/M phases were calculated using Flowjo 10.8.1. The experiment was independently repeated three times, and data were presented as mean ± SEM. Differences between groups were analyzed by one-way ANOVA, with *P* < 0.05 considered statistically significant.

### Tissue microarray (TMA) and immunohistochemistry (IHC)

A TMA containing 89 NPC tissue samples was obtained from SHANGHAI OUTDO BIOTECH CO.,LTD., with the clinical data of these 89 patients provided in Supplementary Data S6; the markers used in this study included ACTBL2 and UNC13D, and the primary antibodies were rabbit anti-human ACTBL2 polyclonal antibody (Cat. No. DF4799, Affinity Biosciences) diluted at 1:200 and mouse anti-human UNC13D monoclonal antibody (Cat. No. 67193-1-Ig, Proteintech) diluted at 1:500, after which tissue sections were scanned using the TissueFAXS SpectraS Automatic Quantitative Pathological Imaging System (Tissue Gnostics); the specific scoring criteria were as follows—the staining status of each tissue spot was observed under low magnification and classified into three grades based on staining intensity: weak positive (1 + , pale yellow), moderate positive (2 + , brownish yellow), and strong positive (3 + , brown); 100 cells were randomly selected in one field of view of each tissue spot to calculate the percentage of positive cells (denoted as X₁%), the same procedure was repeated in another two fields of view to determine the positive cell percentages (denoted as X₂% and X₃%), the average of the three percentages was taken as the final positive rate of the tissue spot, then the H-Score was calculated based on staining intensity and positive rate using the formula: H-Score = (Percentage₀₊ × 0) + (Percentage₁₊ × 1) + (Percentage₂₊ × 2) + (Percentage₃₊ × 3), and the resulting H-Score ranged from 0 to 300, representing the continuous expression level of the target protein.

### Immunofluorescence (IF) analysis

The cells were seeded on coverslips in 24-well plates. After the cells adhered to the well, the culture medium was then replaced with fresh medium containing different concentrations of drugs for 24 h. Then the cells were washed twice with PBS and fixed with 4% paraformaldehyde (Solarbio) at room temperature (RT) for 20 min. Cells were then washed again with PBS and incubated with 0.1% (v/v) Triton X-100 (Sigma) in PBS for 10 min at RT. After washing twice with PBS, cells were blocked in 2% BSA (Sigma) in PBS for 1 h at RT and incubated with primary antibodies (anti-γH2AX, Abcam, ab9110; 1:200 dilution) overnight at 4 °C. After washing five time with PBS, the cells were incubated with secondary antibodies (GeneTex, GTX213111-05) for 1 h and then DAPI (Biosharp) for 5 min at RT, followed by extensive washes with PBS. Next, cells were mounted on a slide with fluoromount-G (SouthernBiotech). Imaging was performed with a Leica DMi8 microscope.

Ten cases of NPC tissues and ten cases of non-cancerous nasopharyngeal tissues and tissue microarrays were processed as follows: tissue microarrays were placed in an oven at 63 °C for 1 h to bake paraffin. Deparaffinization was performed using the LEICA ST50 automatic staining machine. For antigen retrieval, 10 × retrieval solution (AR6001KT, Akoya) was diluted to 1 × working solution; the solution was boiled in a microwave at high power for 3 min, then slides were immersed, and microwave power was adjusted to low power for further retrieval for 15–20 min (ensuring tissues remained submerged throughout). Slides were cooled naturally to room temperature. After retrieval, slides were washed with TBST, placed in a humidified chamber, and incubated with blocking buffer (ARD1001EA, Akoya Biosciences) for 10 min. Primary antibodies (anti-IGF2BP3 mouse monoclonal, MA5-32838, Thermo Fisher Scientific, 1:500; anti-FERMT1 rabbit monoclonal, 22215-1-AP, Proteintech, 1:100; anti-CD33 rabbit monoclonal, ab269456, Abcam, 1:100; anti-CD11B rabbit monoclonal, ab133357, Abcam, 1:100; anti-CD4 rabbit monoclonal, PA285, Abcarta, 1:100; anti-CD8 rabbit monoclonal, PA577, Abcarta, 1:100; Abcarta, 1:100; anti-Foxp3 mouse monoclonal, PA448, Abcarta, 1:100) were added to the humidified chamber and incubated at room temperature for 1 h, followed by TBST washes. Secondary antibodies were then applied and incubated at room temperature for 10 min, with subsequent TBST washes. Opal dye dilution (1:100) was added and incubated at room temperature for 10 min, followed by TBST washes. To remove primary/secondary antibodies, microwave retrieval was performed, and slides were washed with TBST. For multiplex staining, blocking to antibody removal were repeated until all targets were labeled. Finally, DAPI (BDHM-0009, Beida Heming Technology) working solution was applied in a humidified chamber for 10 min at room temperature; slides were washed with TBST, then mounted with VECTASHIELD® HardSet Antifade Mounting Medium (H-1400, Vector Labs). Images were scanned using the TissueFAXS SpectraS automatic quantitative pathological imaging system (Tissue Gnostics). Quantification was performed with Qupath software (version 0.4.3), and statistical analysis was conducted using unpaired Student’s t-test.

### Apoptosis detection by flow cytometry

The cells treated with different concentrations of drugs or control for 72 h were collected and rinsed with cold PBS. Subsequently, the cells were incubated with Annexin V-FITC and PI (556547, BD) in binding buffer for 20 min in the dark. The samples were then analyzed on a flow cytometer. Cells showing Annexin V-positive staining but PI-negative were classified as early apoptotic cells, and those positive for both Annexin V and PI were late apoptotic or necrotic.

### HR (homologous recombination) detection by flow cytometry

Cells were first transfected with the DR-GFP plasmid for 72 h before transfecting with I-Sce plasmid for 24 h. Cells were treated with or without Panobinostat or MYCi975 for 48 h before washing with ice-cold PBS and then subjected to flow cytometry analysis to quantify the proportion of GFP-positive cells.

### Data visualization

Data visualization was performed in R (version 4.2.2), using the ggplot2 (version 3.4.1), ggpubr (version 0.6.0), ggraph (version 2.1.0), pheatmap (version 1.0.12), and UpSetR (version 1.4.0) packages.

## Supplementary information

SI

Data S1

Data S2

Data S3

Data S4

Data S5

Data S6

Data S7

Data S8

Data S9

## Data Availability

The data that support the findings of this study—including clinical information, and proteome and phosphoproteome data—are available within the paper and its Supplementary Information. The raw files of proteome and phosphoproteome datasets can be obtained from iProX database (accession number IPX0011763000).^84^ RNA-seq was deposited in the Gene Expression Omnibus database under accession GSE295454. All other data are available from the corresponding authors upon request. Source data are provided with this paper.
