# Supplementary material for: Large-scale and high-resolution mass spectrometry-based proteomics defines molecular subtypes of nasopharyngeal carcinoma for therapeutic targeting
Source: Signal Transduct Target Ther. 2026 Jun 23;11:244. doi: 10.1038/s41392-026-02742-0 (PMC13287748; doi:10.1038/s41392-026-02742-0)
Supplement: Supplementary file 1 — SI [file 41392_2026_2742_MOESM1_ESM.docx]

Supplementary Materials for

Large-scale and high-resolution mass spectrometry-based proteomics defines molecular subtypes of nasopharyngeal carcinoma for therapeutic targeting

Yi-ping Wu^1, 2, #^, Yao-hui He^3, 4, 6, #^, Guo-sheng Hu^3, 4, 7, #^, Zhi-qin Li^2, #^, Qing-wen Li^1, 2^, Xiao-tong Chen^1, 2^, Hui-ying Ling^1, 2^, Wen Liu^1, 2, 3, 4, 5, *^, Qin Lin^1, 2, *^

Correspondence to: [linqin05@163.com](mailto:linqin05@163.com), [w2liu@xmu.edu.cn](mailto:w2liu@xmu.edu.cn)

**This PDF file includes:**

Figures. S1 to S6

Captions for Data S1 to S9

**Other Supplementary Materials for this manuscript include the following:**

Data S1 to S9

Data S1. The clinical information for the 79 patients with NPC.

Data S2. The expression of the 12,141 proteins quantified with high confidence in at least one of the 10 groups (Prot1).

Data S3. The abundances of the 30,106 phosphosites quantified in at least one of the 10 groups (Phos1).

Data S4. The dysregulated proteins/phosphosites and pathways in NPC.

Data S5. The dysregulated pathways (sheet 1), proteins (sheet 2), and OS (sheet 3) and PFS (sheet 4) of these proteins as shown in Fig. 1f.

Data S6. Subtyping and validation of S1 and S2, as well as the differentially expressed (DE) proteins and their associated pathways between the S1 and S2 subtypes.

Data S7. Drug prediction based on proteomic analysis for NPC.

Data S8. The list of differentially expressed genes (FC > 1.5) between control and Panobinostat-treated HK1 cells and their associated pathways.

Data S9. The list of qPCR primers used in this study. F: forward; R: reverse.


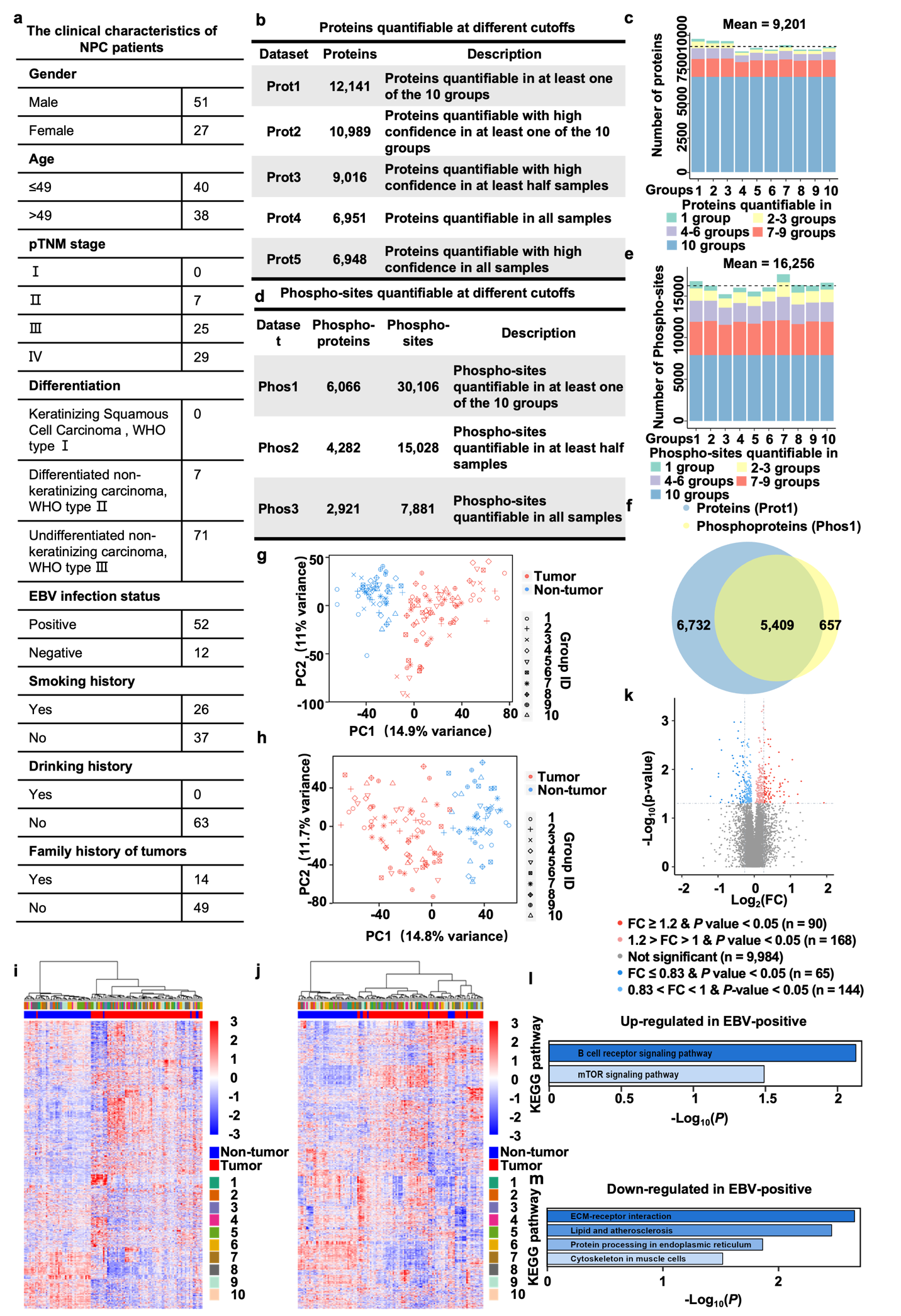
**Figure. S1. Proteomic and phosphoproteomic data description.**

(a) Clinical information of 79 patients with NPC is presented. (b) Proteins detected by proteomic analysis at different cutoffs as indicated. (c) The distribution of the number of groups in which the proteins were quantifiable. A total of 12,141 proteins were identified in all 10 groups. (d) Phosphosites and phosphoproteins detected by phosphoproteomic analysis at different cutoffs as indicated. (e) The distribution of the number of groups in which the phosphosites were quantifiable. A total of 30,106 phosphosites were identified in all 10 groups. (f) The overlap of proteins and phosphoproteins is shown by Venn diagram. Prot1: Proteins quantifiable in at least one of the 10 groups. Phos1: Phosphosites quantifiable in at least one of the 10 groups. (g, h) Principle component analysis (PCA) of the proteomic (g) and phosphoproteomic (h) data separated tumor samples from non-tumor samples, and no batch effects were observed. TMT groups are shown with different shapes. Tumor and non-tumor samples are colored in red and blue, respectively. (i) The hierarchical clustering analysis of the quantifiable proteins was conducted on the 79 NPC tumor and 52 non-tumor samples. (j) Hierarchical clustering analysis of the quantifiable phosphosites was conducted on the 79 NPC tumor and 52 non-tumor samples. (k) Volcano plot showing proteins upregulated or downregulated in the S2 subtype compared to the S1 subtype. Light red and light blue colors represent proteins with *P* < 0.05 and 0.83 < fold change (FC) < 1.2, whereas dark red and dark blue represent proteins with *P* < 0.05 and FC ≥ 1.2 or FC ≤ 0.83. The remaining proteins are depicted in grey. *P* values were calculated using the two-sided Wilcoxon signed-rank test. (l, m) Enriched KEGG pathway for upregulated (l) and downregulated (m) proteins in EBV-positive NPC as shown in (k).


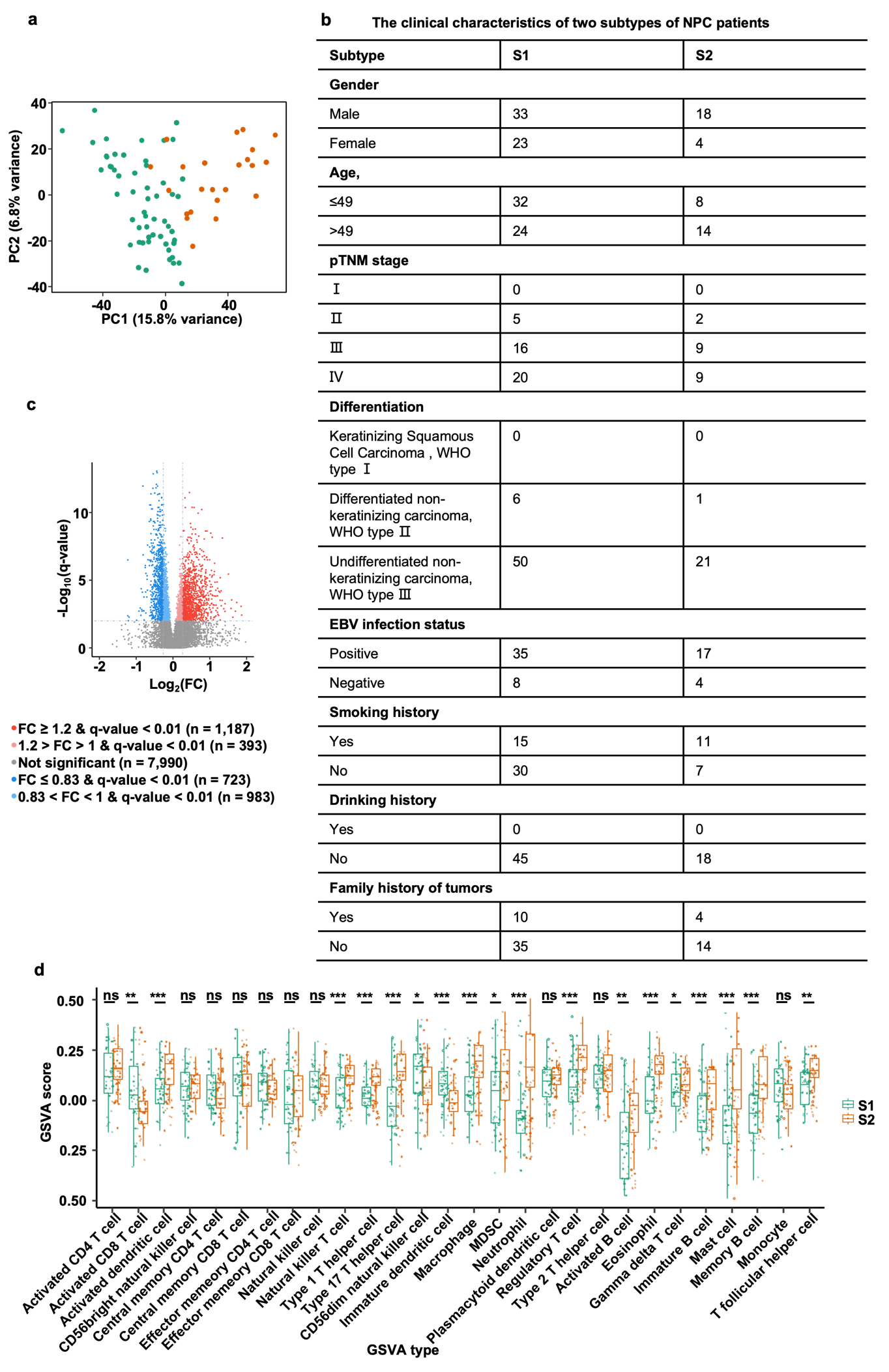

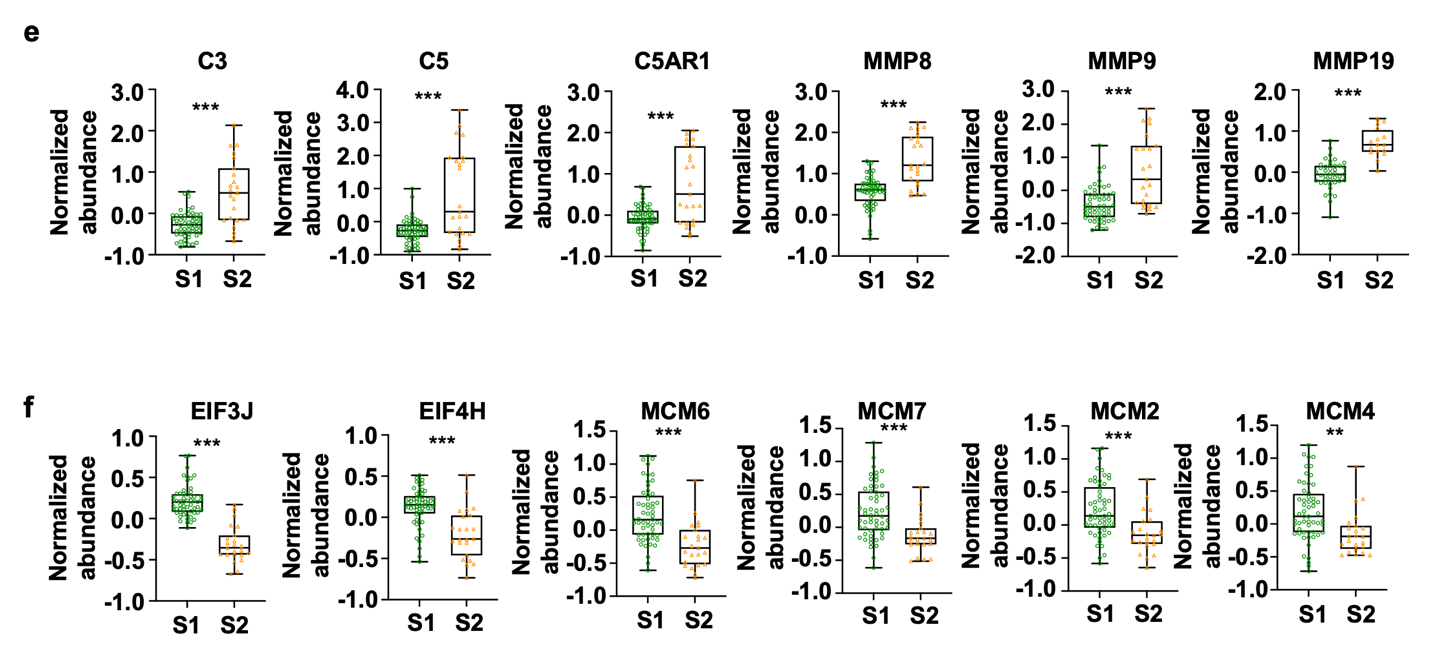


Figure. S2. Representative differentially expressed proteins between S1 and S2 subtypes.

(a) PCA of the proteomic data can separate the two subtypes. S1 and S2 subtypes are colored in green and orange, respectively. (b) Clinical information of 56 patients with NPC of subtype S1 and 23 patients with subtype S2 is shown. (c)Volcano plot showing proteins upregulated or downregulated in the S2 subtype compared to the S1 subtype. Light red and light blue colors represent proteins with Benjamini-Hochberg (BH) adjusted P value < 0.01 and 0.83 < FC < 1.2, whereas dark red and dark blue represent proteins with BH adjusted P value <0.01 and FC ≥ 1.2 or FC ≤ 0.83. The remaining proteins are depicted in grey. BH adjusted P values were calculated using the two-sided Wilcoxon signed-rank test. (c) GSVA enrichment scores across various immune cell types between the S1 and S2 subtypes of NPC are shown. P values were calculated using the two-sided Wilcoxon signed-rank test. (*P < 0.05; **P < 0.001; ***P < 0.001; ns: non-significant). (d, e) The expression of representative proteins in the complement or MYC TARGETs V1 pathway is shown in S1 and S2 subtypes by Box plots (S1, n = 56; S2, n = 23) in proteomic data. P values are calculated by two-sided Wilcoxon signed-rank test. (*P < 0.05; **P < 0.001; ***P < 0.001).


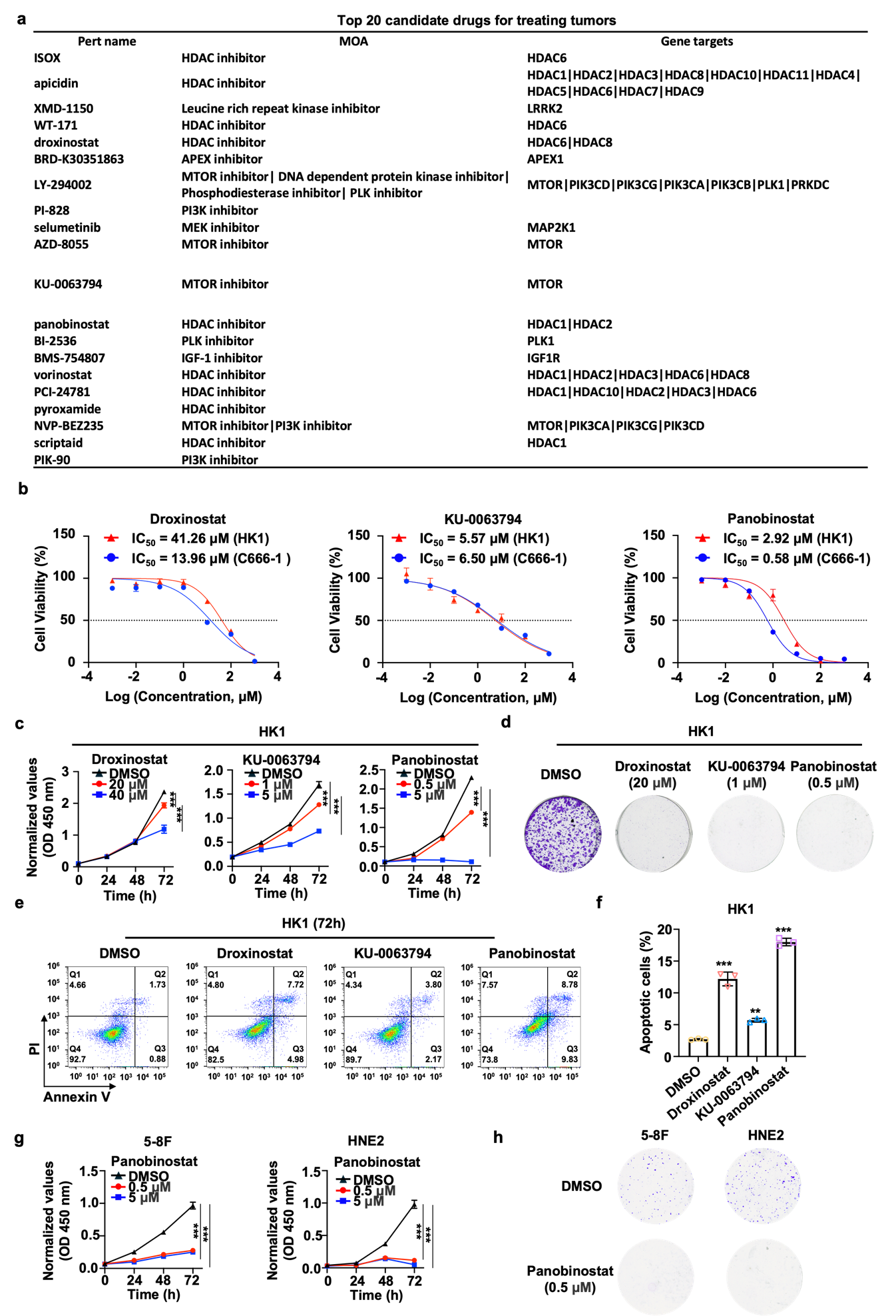

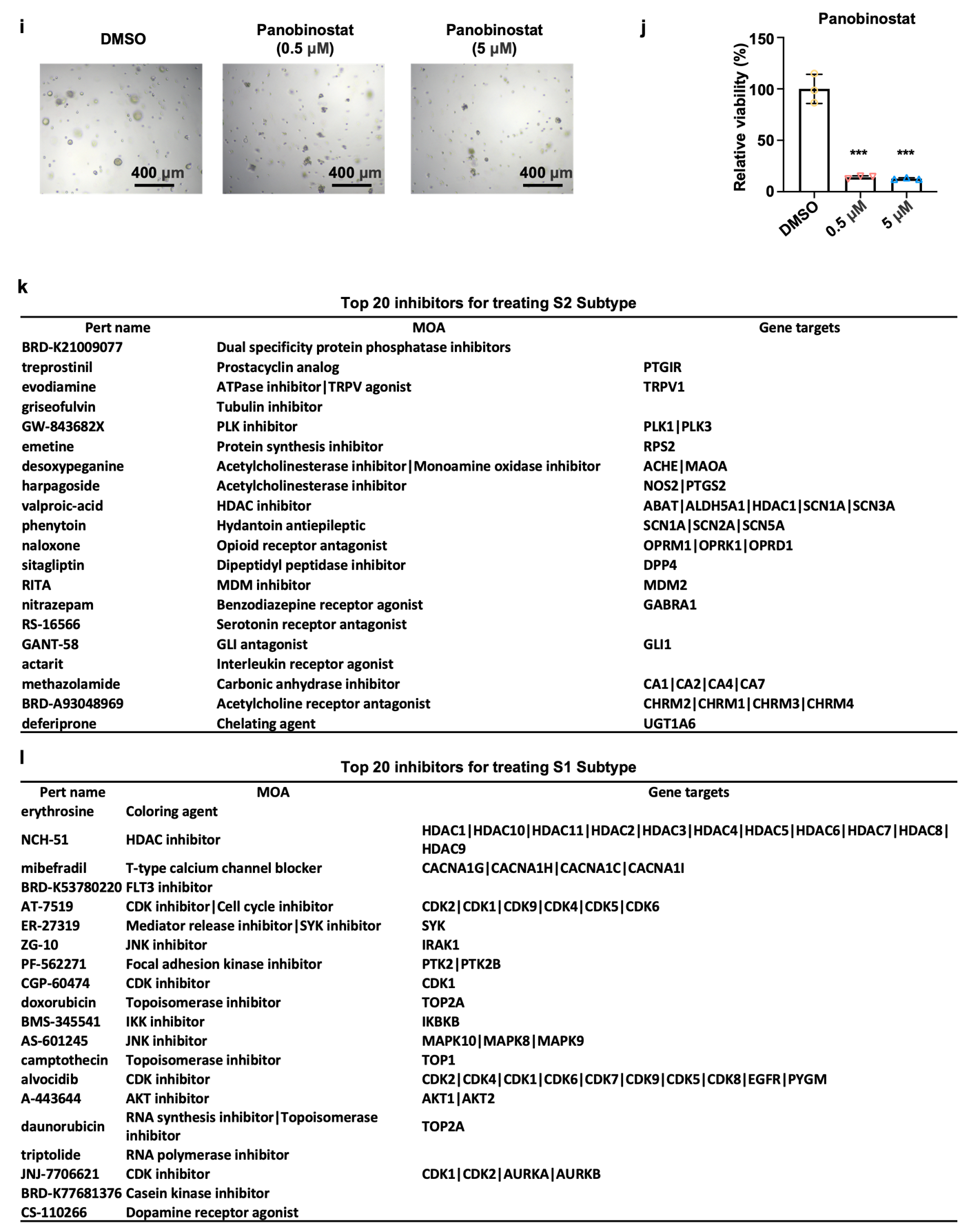


Figure. S3. Drug prediction and validation for NPC.

**(a)** The top 20 perturbagens predicted for NPC are displayed. (b) Cell viability inhibition curves of HK1 and C666-1 NPC cell lines after 48 hours of treatment with three drugs, Droxinostat, KU-0063794, or Panobinostat, are shown. IC₅₀ is indicated. (c) The viability of HK1 cells was assessed by CCK-8 assay after treatment with three drugs at concentrations as indicated for 0, 24, 48 and 72 h (mean ± SEM; ****P* < 0.001). (d) HK1 cells treated with DMSO or three drugs (Droxinostat, 20 mM; KU-0063794, 1 μM; Panobinostat, 0.5 μM) were subjected to colony formation assay for two weeks. (e) HK1 cells were treated with DMSO or three drugs (Droxinostat, 20 μM; KU-0063794, 1 μM; Panobinostat, 0.5 μM) for 72 h, followed by staining with Annexin V-FITC and PI. Apoptotic cells were detected by flow cytometry analysis. (f) Quantification of the apoptotic cells as described in (e) is shown (mean ± SEM; ***P* < 0.01; ****P* < 0.001). (g) The top 20 perturbagens predicted for S2 subtype are displayed. (g) The viability of 5-8F and HNE2 cells was assessed by CCK-8 assay after treatment with three drugs at concentrations as indicated for 0, 24, 48 and 72 h (mean ± SEM; ****P* < 0.001). (h) 5-8F and HNE2 cells treated with DMSO or Panobinostat (0.5 μM) were subjected to colony formation assay. (i) Morphology and size of organoids in response to DMSO or Panobinostat treatment at the indicated concentrations were visualized under bright-field microscopy. Scale bars: 400 μm. (j) The viability of NPC organoids was evaluated via ATP assay following 72 h of treatment with DMSO or Panobinostat at the indicated concentrations (mean ± SEM; ****P* < 0.001). (k, l) The top 20 perturbagens predicted for S2 (k) and S1 (l) subtype are displayed.


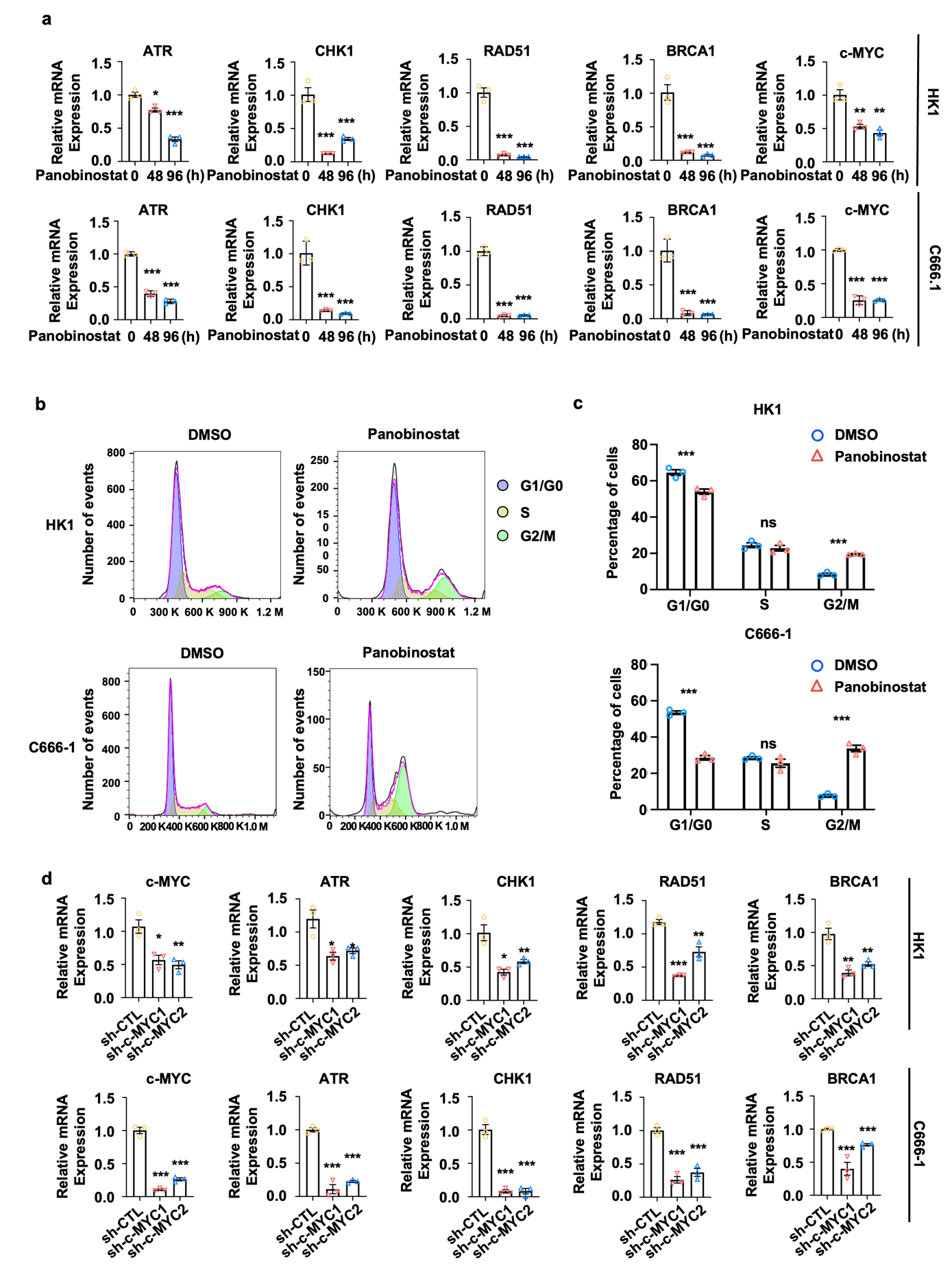


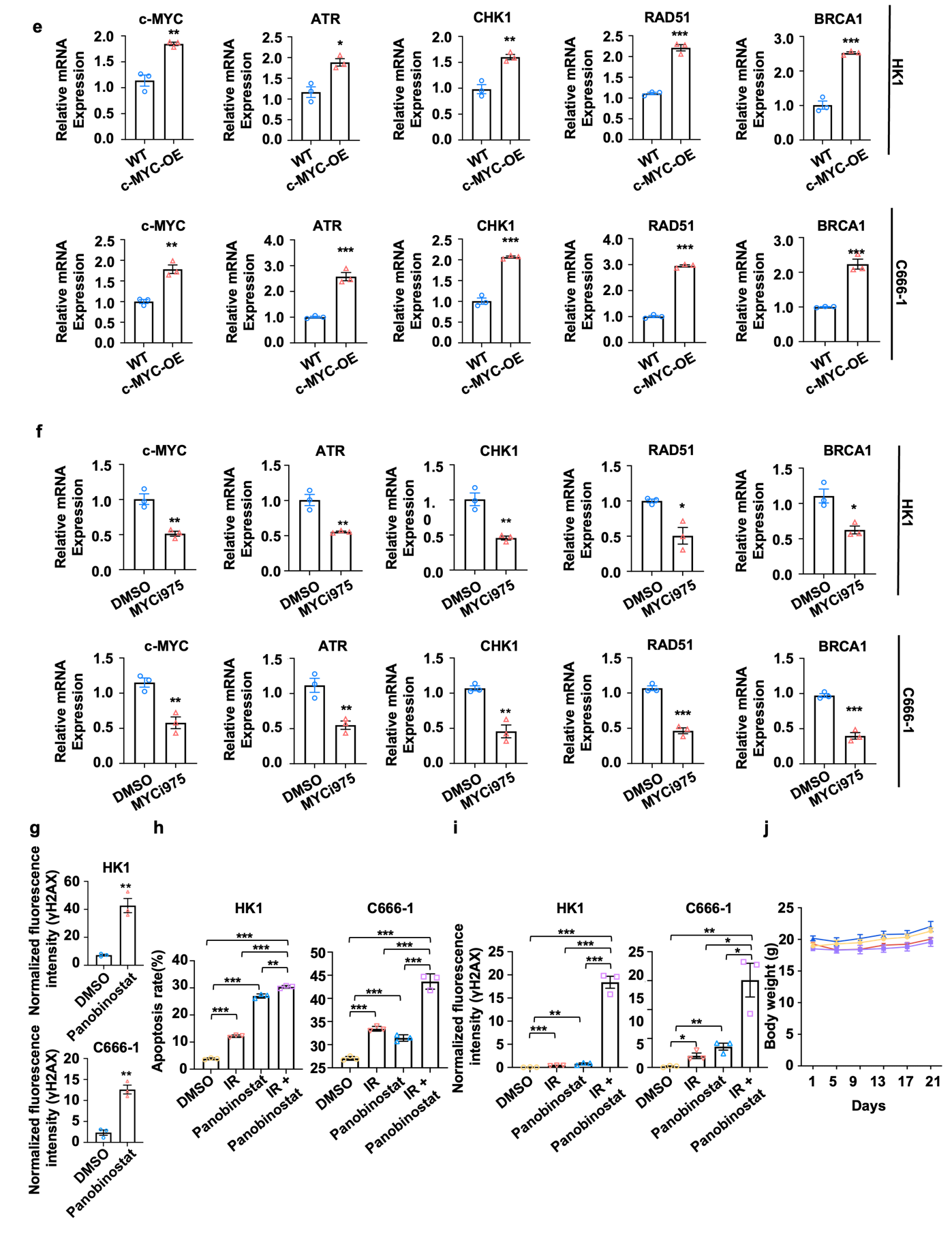


Figure. S4. Panobinostat inhibits HR DNA repair through suppressing MYC-mediated gene transcriptional activation, and combination with IR exhibits synergistic effects.

(a) HK1 and C666-1 cells were treated with Panobinostat (100 nM) for 0, 48, or 96 h, followed by RT-qPCR analysis to examine the expression of genes as indicated (mean ± SEM; **P* < 0.05, ** *P* < 0.01，****P* < 0.001，*****P* < 0.0001). (b) Flow cytometry plots show the cell cycle distribution of HK1 (upper panels) and C666-1 (bottom panels) cell lines after treatment with DMSO or 100 nM Panobinostat for 48 hours. The y-axis represents cell number, and the x-axis represents DNA content. (c) Quantification of the cell cycles distribution as described in (b) is shown (mean ± SEM; ****P* < 0.001). (d) HK1 and C666-1 cells were infected with lentivirus expressing shCTL or two individual sh-c-MYC (sh-c-MYC1 and sh-c-MYC2) for 72 h, followed by RT-qPCR analysis to examine the expression of genes as indicated (mean ± SEM; **P* < 0.05 and ***P* < 0.01，****P* < 0.001). (e) HK1 and C666-1 cells were transfected with control vector or vector expressing c-MYC (c-MYC-OE) for 72 h, followed by RT-qPCR analysis to examine the expression of genes as indicated (mean ± SEM; **P* < 0.05, ***P* < 0.01，****P* < 0.001). (f) HK1 and C666-1 cells were treated with MYCi975 (5 μM) for 24 h, followed by RT-qPCR analysis to examine the expression of genes as indicated (mean ± SEM; **P* < 0.05, ***P* < 0.01，****P* < 0.001). (g) HK1 and C666-1 cells treated with DMSO or Panobinostat (100 nM) for 48 h were subjected to immunostaining with γH2AX antibody. Nuclei are indicated by DAPI. The foci were quantified by Image J (mean ± SEM; ***P* < 0.01). (h) Quantification of the apoptotic cells as described in Fig. 6l is shown (mean ± SEM; ***P* < 0.01, ****P* < 0.001). (i) HK1 and C666-1 cells treated with Panobinostat (50 nM, 48 h) or IR (6 Gy, 12 h) alone or in combination were subjected to immunostaining with γH2AX antibody. Nuclei are indicated by DAPI. The foci were quantified by Image J (mean ± SEM; **P* < 0.05, ***P* < 0.01, ****P* < 0.001). (j) The body weight of mice as described in Fig. 6m is shown (mean ± SEM).


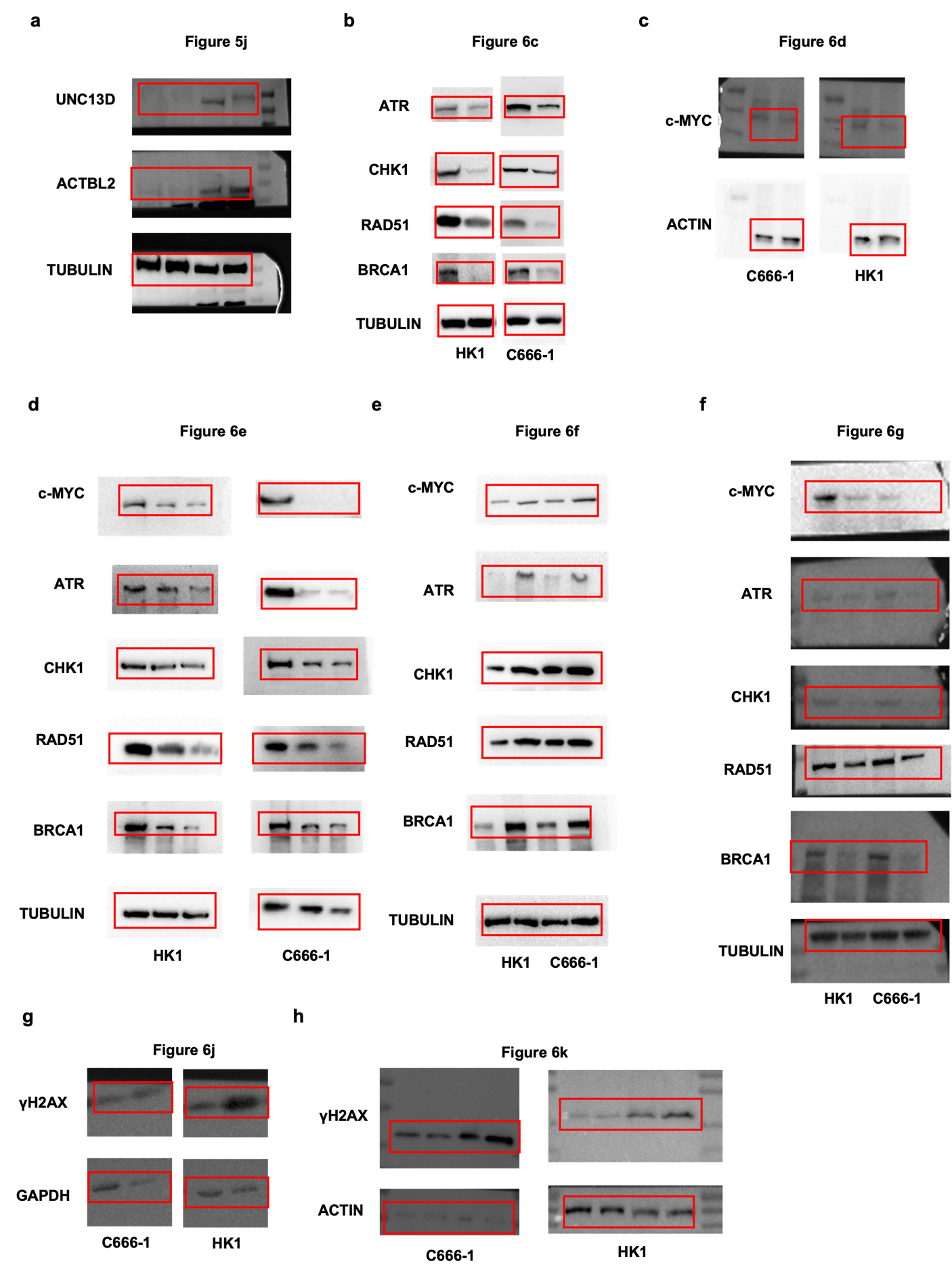


Figure. S5. ALL original and uncropped films of Western blots

(a) Original and uncropped films of Western blots of Fig. 5j. (b) Original and uncropped films of Western blots of Fig. 6c. (c) Original and uncropped films of Western blots of Fig. 6d. (d) Original and uncropped films of Western blots of Fig. 6e. (e) Original and uncropped films of Western blots of Fig. 6f. (f) Original and uncropped films of Western blots of Fig. 6g. (g) Original and uncropped films of Western blots of Fig. 6j. (h) Original and uncropped films of Western blots of Fig. 6k.


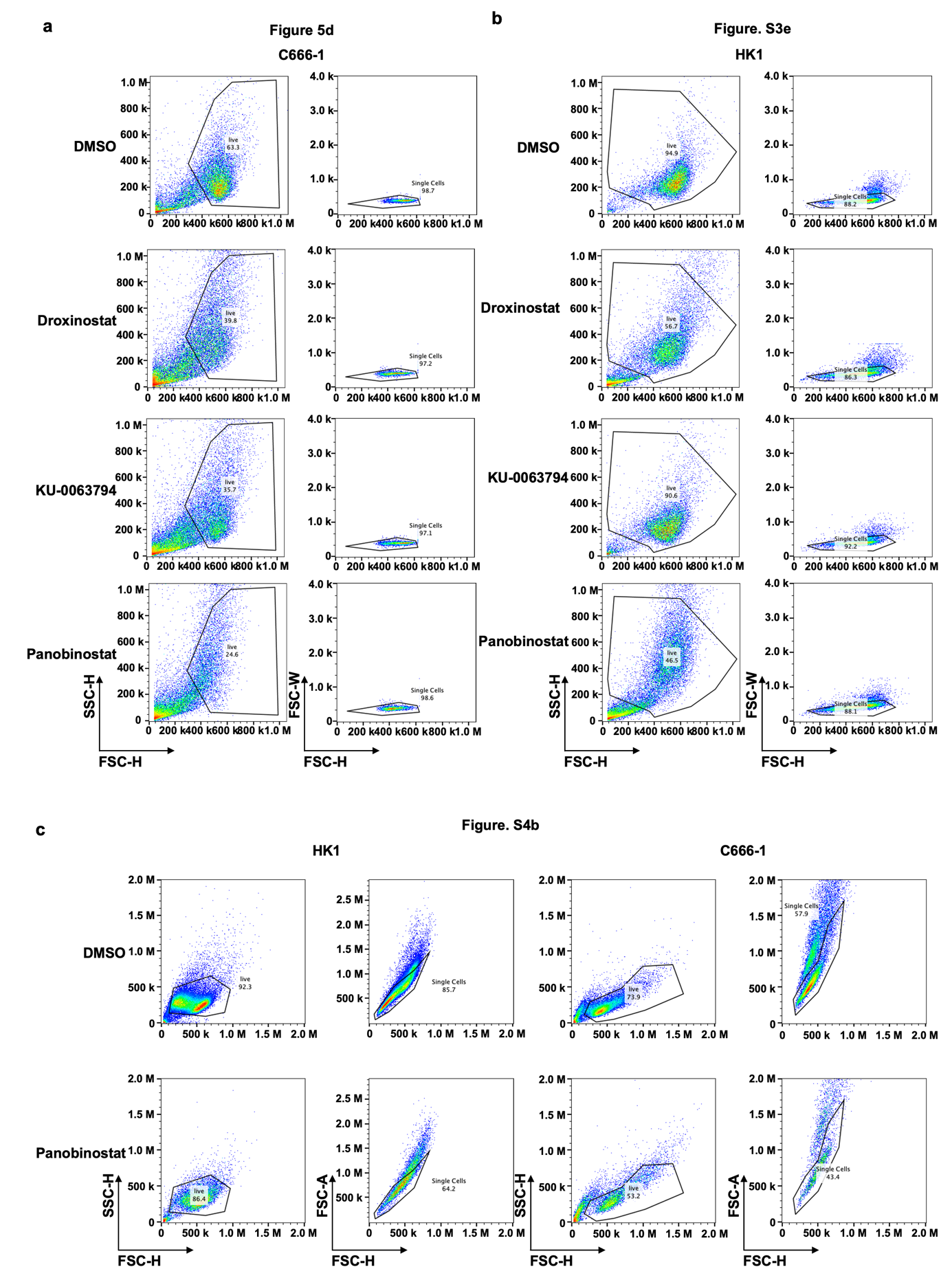


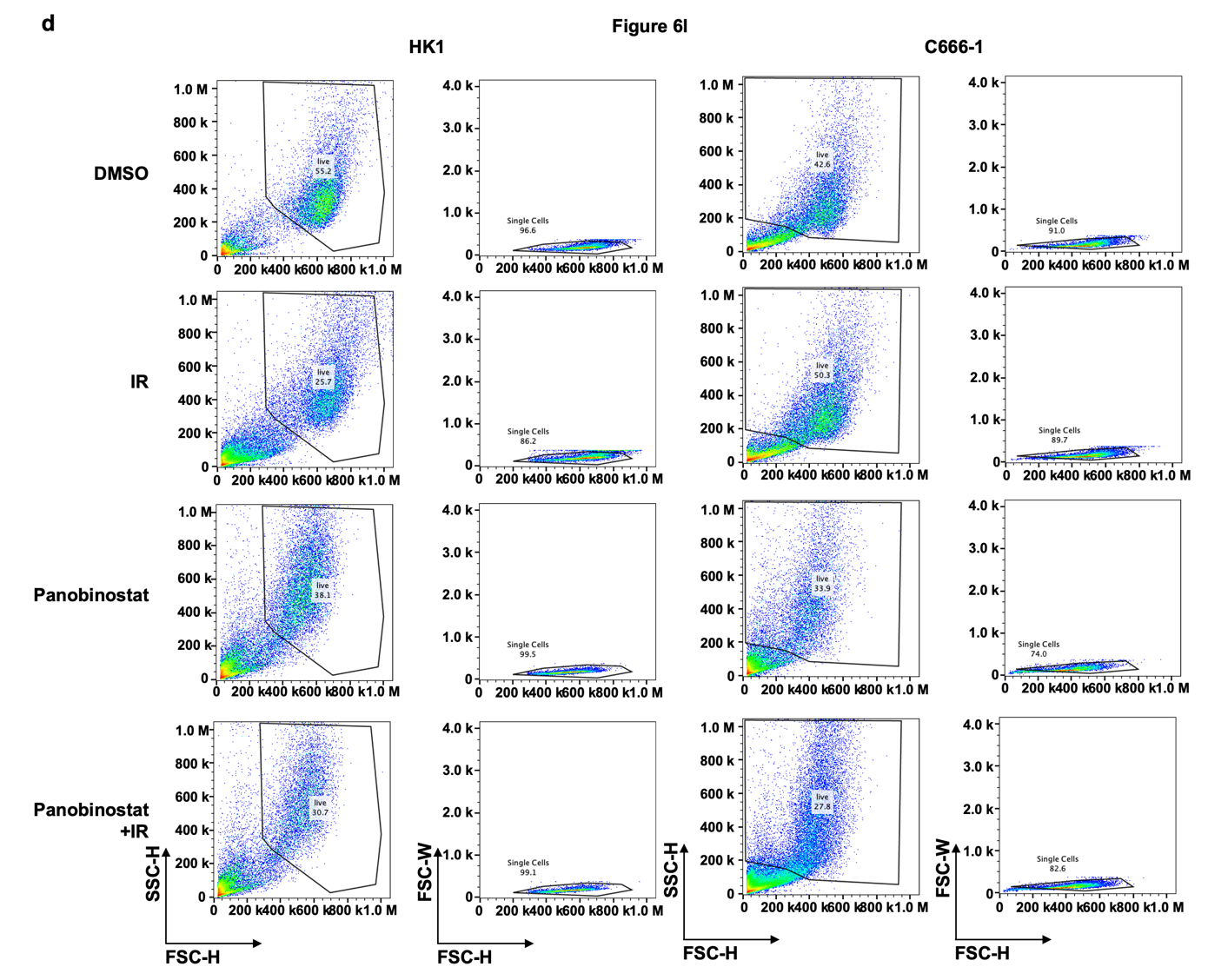


Figure. S6. Gating strategies of Flow cytometry.

(a) Gating strategies of Flow cytometry of Fig. 5d. (b) Gating strategies of Flow cytometry of Fig. S3e. (c) Gating strategies of Flow cytometry of Fig. S4b. (d) Gating strategies of Flow cytometry of Fig. 6l.

Data S1. The clinical information for the 79 patients with NPC.

Data S2. The expression of the 12,141 proteins quantified with high confidence in at least one of the 10 groups (Prot1).

The values were subjected to mean normalization by column and log_2_ transformation.

Data S3. The abundances of the 30,106 phosphosites quantified in at least one of the 10 groups (Phos1).

Data S4. The dysregulated proteins/phosphosites and pathways in NPC.

Sheet 1: Description; Sheet 2 and 4: Dysregulated proteins (sheet 2) and pathways (sheet 4) between tumor and non-tumor samples; Sheet 3 and 5: Dysregulated phosphosites (sheet 3) and pathways (sheet 5) between tumor and non-tumor samples.

Data S5. The dysregulated pathways (sheet 1), proteins (sheet 2), and OS (sheet 3) and PFS (sheet 4) of these proteins as shown in Fig. 1f.

Data S6. Subtyping and validation of S1 and S2, as well as the differentially expressed (DE) proteins and their associated pathways between the S1 and S2 subtypes.

Sheet 1: Description; Sheet 2: The molecular subtype of the 79 patients with NPC; Sheet 3-10: The differentially expressed proteins (sheet 3, 5, 6, and 7) and associated pathways (sheet 4, 8, 9, and 10) between S1, S2 and/or non-tumor samples. Sheet 11: Subtype classification information and clinical information of NPC patients on the tissue microarray. Sheet 12: The H-score of ACTBL2 and UNC13D based on immunohistochemical staining on the tissue microarray.

Data S7. Drug prediction based on proteomic analysis for NPC.

Sheet 1: Description. Sheet 2: The list of the top 150 up-regulated and down-regulated proteins in tumor compared to non-tumor samples that used as the query signature for CMAP. Sheet 3: Drugs predicted for NPC based on proteins as shown in sheet 2. Sheet 4: The list of the top 150 up-regulated and down-regulated proteins in S2 compared to S1 that used as the query signature for CMAP. Sheet 5: Drugs predicted for subtype 1 and 2 based on proteins as shown in sheet 4.

Data S8. The list of differentially expressed genes (FC > 1.5) between control and Panobinostat-treated HK1 cells and their associated pathways.

Data S9. The list of qPCR primers used in this study. F: forward; R: reverse.
